# NAMPT orchestrates fibroblast cuproptosis and immune crosstalk during IPF progression

**DOI:** 10.3389/fimmu.2026.1726692

**Published:** 2026-05-20

**Authors:** Junyu Jiang, Xinghe Liu, Guo Yang, Jia Xie, Weijiao Mou, Yunfei Xiang, Tao Zhang, Wenyu Du, Qingsong Chen, Fating Zhou, Guangbin Huang, Dingyuan Du

**Affiliations:** 1 Department of Trauma Surgery, Chongqing Key Laboratory of Emergency Medicine, Chongqing Emergency Medical Center, Central Hospital, School of Medicine, Chongqing University, Chongqing, China; 2 Department of Cardiology, Chongqing Emergency Medical Center, Chongqing, China; 3 Department of Endocrinology, Chongqing Emergency Medical Center, Chongqing, China

**Keywords:** cell-cell communication, cuproptosis, idiopathic pulmonary fibrosis, immune crosstalk, multi-omics, NAMPT

## Abstract

**Background:**

Idiopathic pulmonary fibrosis (IPF) is a chronic, progressive interstitial lung disease characterized by excessive fibroblast activation and extracellular matrix deposition, leading to severe tissue remodeling and poor prognosis. While current treatments offer limited efficacy. Emerging evidence suggests cuproptosis, a novel form of copper-dependent cell death, as a potential mechanism influencing fibroblast survival and fibrogenesis.

**Methods:**

Bulk RNA sequencing (RNA-seq), single-cell RNA sequencing (scRNA-seq), and spatial transcriptomics were utilized to analyze gene expression profiles from IPF patient samples and healthy controls. Differentially expressed genes related to copper regulation and cell death pathways were identified using machine learning algorithms. Immune infiltration was assessed using CIBERSORT, and cell-cell interactions were explored using the CellChat algorithm. Gene set variation analysis (GSVA) and gene set enrichment analysis (GSEA) were employed to explore the role of nicotinamide phosphoribosyltransferase (NAMPT) in cuproptosis and fibrosis-related pathways.

**Results:**

Transcriptomic analysis identified NAMPT as a key hub gene in IPF, with strong positive correlation to cuproptosis. Spatial transcriptomics revealed elevated cuproptosis activity in NAMPT+ fibroblasts localized in fibrotic lesions. Additionally, NAMPT+ fibroblasts exhibited increased crosstalk with M2 macrophages through the C3-ITGB2 complement pathway, implicating NAMPT in promoting fibroblast activation and shaping a pro-fibrotic immune microenvironment.

**Conclusion:**

This study identifies NAMPT as a critical regulator of cuproptosis in IPF fibroblasts and highlights its dual role in fibroblast activation and immune modulation. Targeting NAMPT offers a promising therapeutic strategy for modulating fibroblast behavior, reducing fibrosis, and disrupting the pro-fibrotic cell communication network in IPF.

## Introduction

Idiopathic pulmonary fibrosis (IPF) is a chronic and progressive interstitial lung disease, characterized by excessive extracellular matrix (ECM) deposition and persistent fibroblast activation, which leads to a decline in pulmonary gas exchange and ultimately respiratory failure (1–3). IPF typically carries a poor prognosis, with an extremely low 5-year survival rate (4, 5). During IPF progression, a variety of cell types, including epithelial cells, fibroblasts, and endothelial cells, play key roles in disease development (6–8). Additionally, the complex immune microenvironment of IPF is a significant factor that complicates treatment efforts. This microenvironment involves diverse intercellular crosstalk within fibrotic foci, and recent studies have shown that such interactions are linked to various modes of cell death, including apoptosis, ferroptosis, and pyroptosis (9–11). Despite extensive efforts to uncover the molecular mechanisms underlying IPF, its pathogenesis remains only partially understood, and current approved drugs, such as nintedanib and pirfenidone have limited effectiveness (12). Understanding the molecular pathways that regulate fibroblast activation and tissue remodeling in IPF is essential for developing more effective therapies.

In recent years, a novel form of programmed cell death known as cuproptosis has garnered significant attention. This form of cell death is primarily driven by the accumulation of copper ions and the aggregation of mitochondrial proteins, leading to cytotoxicity and cell death (13). Copper is an essential trace element that plays a role in various biological processes, including extracellular matrix (ECM) remodeling through lysyl oxidase—a copper-dependent enzyme crucial for collagen cross-linking (14). Given copper’s importance in fibrotic tissue remodeling, the potential role of cuproptosis in IPF pathogenesis warrants further investigation. However, the exact involvement of cuproptosis in fibroblast function and the progression of IPF remains to be fully understood.

To investigate the potential role of cuproptosis in IPF, we performed a comprehensive transcriptomic analysis to identify key regulatory genes involved in copper homeostasis and cell death pathways. Bulk RNA sequencing (RNA-seq), single-cell RNA sequencing (scRNA-seq), and spatial transcriptomics were used to examine gene expression profiles in lung tissues from IPF patients and healthy controls. Differential expression analysis, combined with machine learning-based feature selection, identified several genes associated with copper regulation and cell death as potential modulators of fibroblast activity in IPF. Among these, nicotinamide phosphoribosyltransferase (NAMPT) emerged as a key gene of interest.

NAMPT, an enzyme essential for NAD+ biosynthesis, plays a crucial role in various cellular processes, including immune regulatory, inflammation, and cell survival (15–17). Recently, Chen et al. reported that NAMPT prompts bleomycin-induced pulmonary fibrosis by driving macrophage M2 polarization (18), and Zhan et al. revealed that NAMPT facilitates macrophage-mediated pulmonary fibrosis through the Sirt1-Smad7 pathway (19). Also, Garcia’s study has shown that blood NAMPT exacerbate liver steatohepatitis and liver fibrosis via TLR4 inflammatory signaling (20). Above all, NAMPT exhibited its enhancer potential in fibrotic disease. However, the link between NAMPT and cuproptosis, particularly in IPF fibroblasts, remains unexplored. This study aims to investigate the role of NAMPT in modulating cuproptosis in IPF fibroblasts and evaluate its potential contribution to disease progression.

By integrating transcriptomic data with spatial transcriptomics and functional assays, we hypothesize that NAMPT plays a critical role in regulating fibroblast survival through cuproptosis pathways, thereby influencing the progression of fibrosis in IPF. The findings of this study may offer valuable insights into the mechanisms underlying fibroblast dysfunction in IPF and identify NAMPT as a potential therapeutic target for modulating copper-dependent cell death in fibrotic lung disease.

## Methods

### Study design

**Figure 1:**
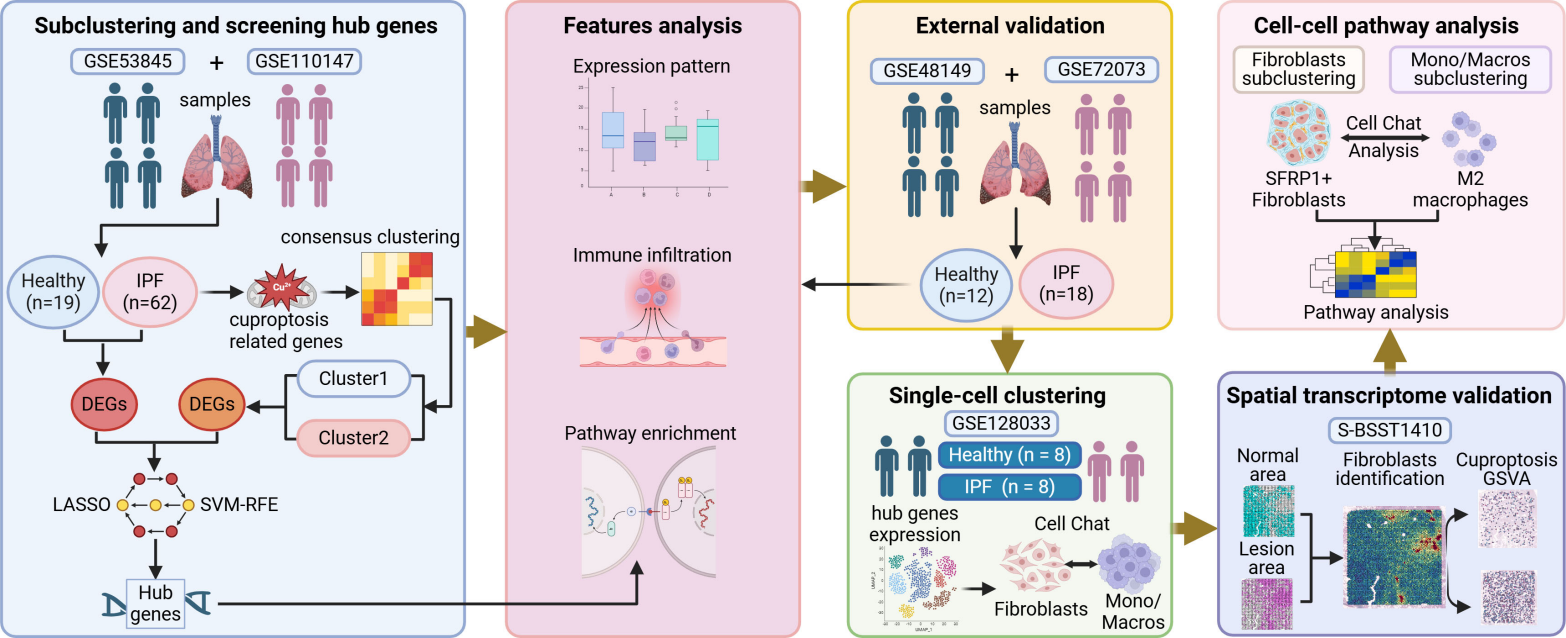
Flow chart of the bioinformatic analysis. Created with Biorender.com.

The study design is illustrated in **Figure 1**.

### Bulk RNA sequencing data collection

Two RNA-seq datasets, GSE53845 and GSE110147, were obtained from the Gene Expression Omnibus (GEO) database. The GSE53845 dataset, annotated using the GPL6480 platform, contained 40 samples from idiopathic pulmonary fibrosis (IPF) patients and 8 from healthy controls. Similarly, GSE110147, annotated with the GPL6244 platform, included 22 IPF samples and 11 controls. Prior to batch correction, expression data were normalized and transformed to approximate continuous distributions, making them suitable for ComBat adjustment. The GSE53845 and GSE110147 datasets were then integrated using the “ComBat” function in the “sva” R package to correct batch effects. The corrected dataset was subsequently used to identify IPF subtypes based on the expression of cuproptosis-related genes (CRGs). To quantitatively evaluate batch effect removal, principal component analysis (PCA) and permutational multivariate analysis of variance (PERMANOVA) were performed before and after ComBat adjustment to estimate the relative contributions of dataset and disease status to the overall expression variance. For external validation, datasets GSE72073 and GSE48149, containing 18 IPF samples and 12 healthy controls, were utilized and annotated with the GPL17586 and GPL16221 platforms, respectively.

### Differential expression genes analysis

To identify significantly differentially expressed genes (DEGs) and investigate molecular characteristics within IPF datasets, we utilized the “Limma” R package, a tool specifically designed for analyzing differential gene expression. The DEGs were determined by contrasting IPF samples with control samples and assessing subtypes derived from CRG expression patterns. Genes were selected for further exploration if they met the criteria of a p-value < 0.05 and |log2FC| > 0.5. These DEGs were displayed in a volcano plot created with the “ggplot2” R package, showcasing their distribution.

### Consensus clustering analysis

In the initial analysis, the human bulk RNA-seq datasets GSE53845 and GSE110147 were integrated. CRGs were obtained from a previous study (21). Then, consensus clustering was performed using the “ConsensusClusterPlus” package, with samples grouped according to CRGs expression patterns and the process 100 iterations. The optimal number of clusters was determined by analyzing the cumulative distribution function (CDF) curve and the consensus matrix heatmap for distinct features. Robustness of consensus clustering has been assessed by leave-one-gene-out sensitivity analysis based on the 19 CRGs. One CRG has been omitted in each iteration, and clustering has been repeated using the same parameter settings as in the main analysis. Similarity between the perturbed and original classifications has been quantified using the adjusted Rand index (ARI).

### Machine learning for identifying CRG-associated hub genes in IPF

To identify hub genes linked to CRGs across various IPF subtypes, we applied LASSO regression and SVM-RFE algorithms. The gene set originated from the overlap of DEGs identified in clusters and comparisons between IPF patients and healthy controls. The “glmnet” package facilitated the implementation of the LASSO method, while the SVM-RFE approach executed recursive feature elimination. SVM modeling employed the “e1071” and “mswmRFE.R” packages to perform reverse feature selection and prioritize hub genes with the highest correlations. All overlapping genes were incorporated into the SVM framework, and the output was visualized. For LASSO, ten-fold cross-validation was performed to select optimal λ values (λ.min and λ.1se), and genes with non-zero coefficients at both thresholds were retained. For SVM-RFE, recursive feature elimination with ten-fold cross-validation was performed to select top features, and feature stability was further evaluated using “FeatSweep” analysis. Stability of hub gene selection has been evaluated by repeated resampling analyses for both LASSO and SVM-RFE (100 runs for each method). For each candidate gene, selection probability and inter-method dispersion have been calculated on the basis of the corresponding selection frequencies across methods.

### Immune infiltration analysis

CIBERSORT was utilized to analyze the relative composition of immune cell types within each sample by deconvolving gene expression data. The LM22 signature matrix, which includes markers for 22 immune cell types, was applied, and 1,000 iterations were performed to quantify immune populations. Samples with a CIBERSORT p-value less than 0.05 were retained for further analysis. Spearman’s nonparametric correlation was employed to assess the relationship between immune cell infiltration and hub genes. Furthermore, the MCP-counter algorithm, implemented through the “MCPcounter” R package, estimated the absolute levels of eight immune cell types and two matrix components (22).

### Gene set enrichment analysis

Gene set enrichment analysis (GSEA) was performed to evaluate differences in signaling pathways among groups exhibiting distinct expression patterns. Gene sets for the analysis were derived from the Molecular Signature Database (MsigDB) and applied to detect pathway distinctions between groups and clusters. Pathways with p-values under 0.05 were prioritized according to their concordance rankings.

### Gene set variation analysis

Gene set variation analysis (GSVA) is a nonparametric, unsupervised method used to evaluate gene set enrichment in transcriptomic datasets. By assigning scores to predefined gene sets, it converts gene-level changes into pathway-level alterations, enabling the evaluation of biological functions within individual samples. In this study, we employed the GSVA algorithm with the Molecular Signature Database R package (msigdbr) version 7.5.1 to analyze potential functional differences among various samples. For consistency, the same parameters (method=“ssgsea”, kcdf=“Gaussian”) were applied across all platforms to ensure comparability.

### Single−cell RNA−seq analysis

#### Database, preprocessing, and integration

A total of 67,622 cells from the GEO database (accession number GSE128033) were analyzed, including eight healthy controls and eight IPF samples sequenced using the Illumina NextSeq 5000 system (23). For downstream processing, Seurat (v5.0.1) was employed. Genes expressed in fewer than three cells were excluded, and cells with fewer than 500 or exceeding 5,000 unique molecular identifiers (UMIs) or with over 30% mitochondrial gene expression were filtered out to reduce potential biases from damaged cells. The mitochondrial gene threshold was determined based on the distribution of percent.MT across cells and visual inspection of quality control metrics, with consideration of the elevated mitochondrial expression commonly observed in lung tissue under pathological conditions. Following this filtering process, 49,986 cells were retained for subsequent analyses. The gene expression data underwent log-normalization, scaling to a maximum of 10,000, and adjustment for total UMIs via the ‘ScaleData’ function. Principal component analysis (PCA) was utilized to reduce the dimensionality of the highly variable genes. Subtypes were then grouped based on the first principal components.

### Clustering and annotation

Clusters were identified within the scRNA dataset at a resolution of 0.4, as determined through the “clustertree” (v0.5.1) tool. To pinpoint conserved marker genes for each cluster, the “FindAllMarkers” function was utilized. Subsequent annotation of clusters was conducted based on their canonical marker genes. Further classification of monocytes and macrophages (Mono/Macros) was achieved by evaluating the expression levels of monocyte markers (LY86, VCAN, ITGAX), M1-specific genes (IL1A, CAMP, MT1H), and M2-associated markers (SPP1, HMOX1, TREM2). The marker genes obtained from online websites, including, cellmarker (http://117.50.127.228/CellMarker/) and cell taxonomy (https://ngdc.cncb.ac.cn/celltaxonomy/).

### Cell−cell communication

CellChat (v2.1.2) was employed with its default parameter settings to explore intercellular communication within the integrated dataset (24). This tool infers cell-to-cell signaling interactions based on a curated database of ligand-receptor pairs. Special focus was given to the interactions involving fibroblasts and immune cells.

### Spatial transcriptome validation

Spatial transcriptomic data for IPF were sourced from the BioStudies repository (https://www.ebi.ac.uk/biostudies/studies/) with the accession number S-BSST1410. Analysis and visualization of feature-barcode matrices were carried out using Seurat 5.0.1. The SCTransform function was applied to normalize the expression matrix. Fibrotic lesions and normal regions were differentiated based on the expression of fibrosis-associated genes, such as COL1A1. Fibroblast-enriched spots were identified through the expression of the cell marker ACTA2. The proportion of NAMPT-positive fibroblasts (spots co-expressing ACTA2 and NAMPT) was quantified in both fibrotic and normal areas. Furthermore, gene set variation analysis was performed to compare fibroblasts with positive and negative NAMPT expression.

### Cell culture and treatment

MRC-5 human embryonic lung fibroblasts (TCH-C263) were obtained from HyCyte Co. Ltd. (China). The cells were cultured in Dulbecco’s modified Eagle’s medium (DMEM) supplemented with 10% fetal bovine serum and 1% penicillin–streptomycin. NAMPT overexpression was induced by transfecting the cells with a plasmid supplied by Gencefebio (China) using Lipo8000™ transfection reagent (Beyotime, C0533).

### Protein extraction and western blotting analysis

Total cellular proteins were isolated using radio immunoprecipitation assay (RIPA) buffer supplemented with phenylmethanesulfonyl fluoride (PMSF) (Beyotime, P1045). Western blot analysis was performed to evaluate relative protein expression. Briefly, denatured proteins were separated via polyacrylamide gel electrophoresis and transferred onto polyvinylidene fluoride (PVDF) membranes. Membranes were blocked with 5% non-fat milk, incubated overnight at 4 °C with primary antibodies, and then probed with horseradish peroxidase-conjugated secondary antibodies. The primary antibodies used included: NAMPT (Proteintech, 11776-1-AP), FDX1 (Abmart, T510671F), CTR1/SLC31A1 (Abmart, T510261F), HSP70 (Abmart, M20033F), β-tubulin (Abmart, M20005F), and anti-Flag (Abmart, M20008F).

### Statistical analysis

Statistical analyses were conducted using R software (version 4.3.3). Group differences were evaluated using either Student’s t-test or the mann-whitney u-test, depending on the distribution of the data. Relationships between variables were assessed through Pearson or Spearman correlation coefficients. A threshold of P < 0.05 was used to define statistical significance, with significance levels categorized as follows: *P < 0.05; **P < 0.01; ***P < 0.001; ****P < 0.0001.

## Results

### Feature and functional alteration in IPF

**Figure 2:**
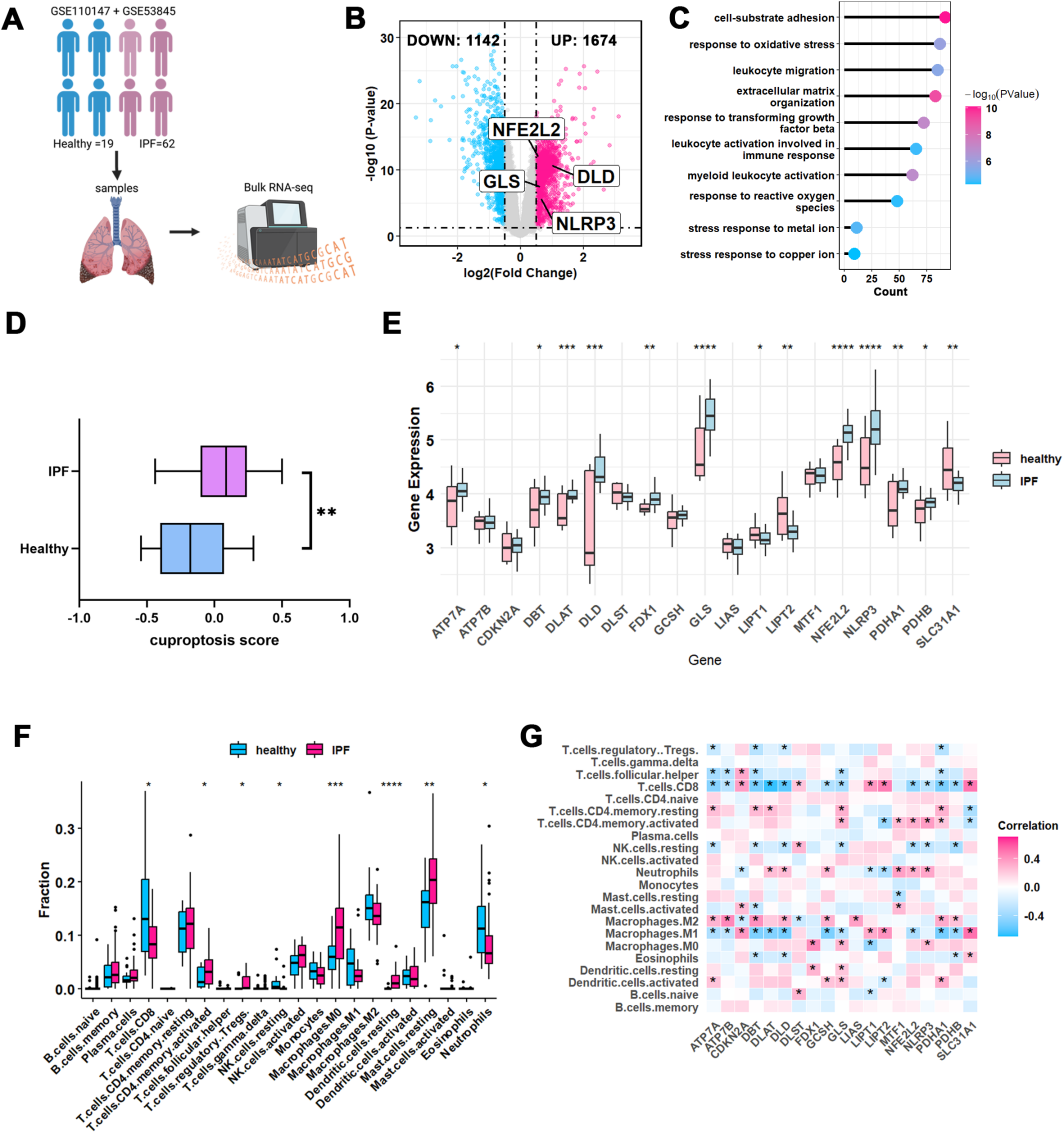
CRGs expression in IPF and healthy control samples. **(A)** Principal component analysis illustrated the comparability of integrated bulk RNA-seq datasets. **(B)** Volcano plot showing differentially expressed genes (DEGs) of IPF *vs.* healthy control, red dots represent up-regulated DEGs and blue dots represent down-regulated DEGs. CRGs are also labeled. **(C)** The lollipop graph exhibits the GO enriched pathways of IPF *vs.* healthy control. **(D)** GSVA score of cuproptosis were visualized in bar plot. **(E)** Expression pattern of 19 CRGs were presented as box plot with mean ± SD. **(F)** Immune infiltration of IPF *vs.* healthy control were assessed via CIBERSORT, and visualized as box plot. **(G)** The correlation analysis between 19 CRGs and immune cells. The color bar indicates the Pearson correlation coefficient. Red represents positive correlation, blue represents negative correlation, and the depth of the color represents the strength of the correlation. *P-values* in the box plots are denoted by asterisks: *P<0.05. CRGs, Cuproptosis-Related Genes; IPF, Idiopathic Pulmonary Fibrosis; GO, Gene Ontology; GSVA, Gene Set Variation Analysis.

Firstly, PCA analysis showed that ComBat adjustment reduced platform-associated variation and improved separation by disease status. Permutational multivariate analysis of variance (PERMANOVA) further demonstrated that dataset effects accounted for most of the variance before correction (R² = 0.988, p = 0.001), whereas the variance explained by dataset was markedly reduced after ComBat adjustment (R² = 0.012, p = 0.358). In contrast, disease status remained significantly associated with expression variation after correction (R² = 0.246, p = 0.001), supporting the validity of downstream consensus clustering and DEG identification (**Supplementary Figure 1**). Then, we analyzed the expression profiles of 81 patients, including 62 IPF patients and 19 healthy controls. (**Figure 2A**). A total of 2,816 differentially expressed genes (DEGs) were identified, with 1,674 genes upregulated and 1,142 downregulated in the IPF group (**Figure 2B**). Gene Ontology enrichment analysis revealed that the upregulated genes in IPF were primarily involved in tissue remodeling and leukocyte immune function (**Figure 2C**).

### CRGs expression and immune infiltration analysis in IPF

To further explore the role of cuproptosis in IPF, cuproptosis-related genes (CRGs) were obtained from a previous study (21). GSVA analysis revealed a significant elevation of cuproptosis in IPF samples (**Figure 2D**). Additionally, several CRGs, including NFE2L2, NLRP3, GLS, and DLD, showed differential expression between IPF patients and healthy controls (**Figure 2E**). Since DEGs in IPF are predominantly enriched in leukocyte immune regulatory functions (**Figure 2C**), we conducted CIBERSORT analysis to assess immune cell infiltration. Compared to healthy controls, IPF samples demonstrated notable immune infiltration, particularly involving macrophages, dendritic cells and T cells (**Figure 2F**). Correlation analysis between CRGs and immune cells showed that most CRGs were positively associated with M2 macrophages, while negatively correlated with M1 macrophages and CD8+ T cells (**Figure 2G**).

### Consensus clustering IPF based on expression of CRGs

**Figure 3:**
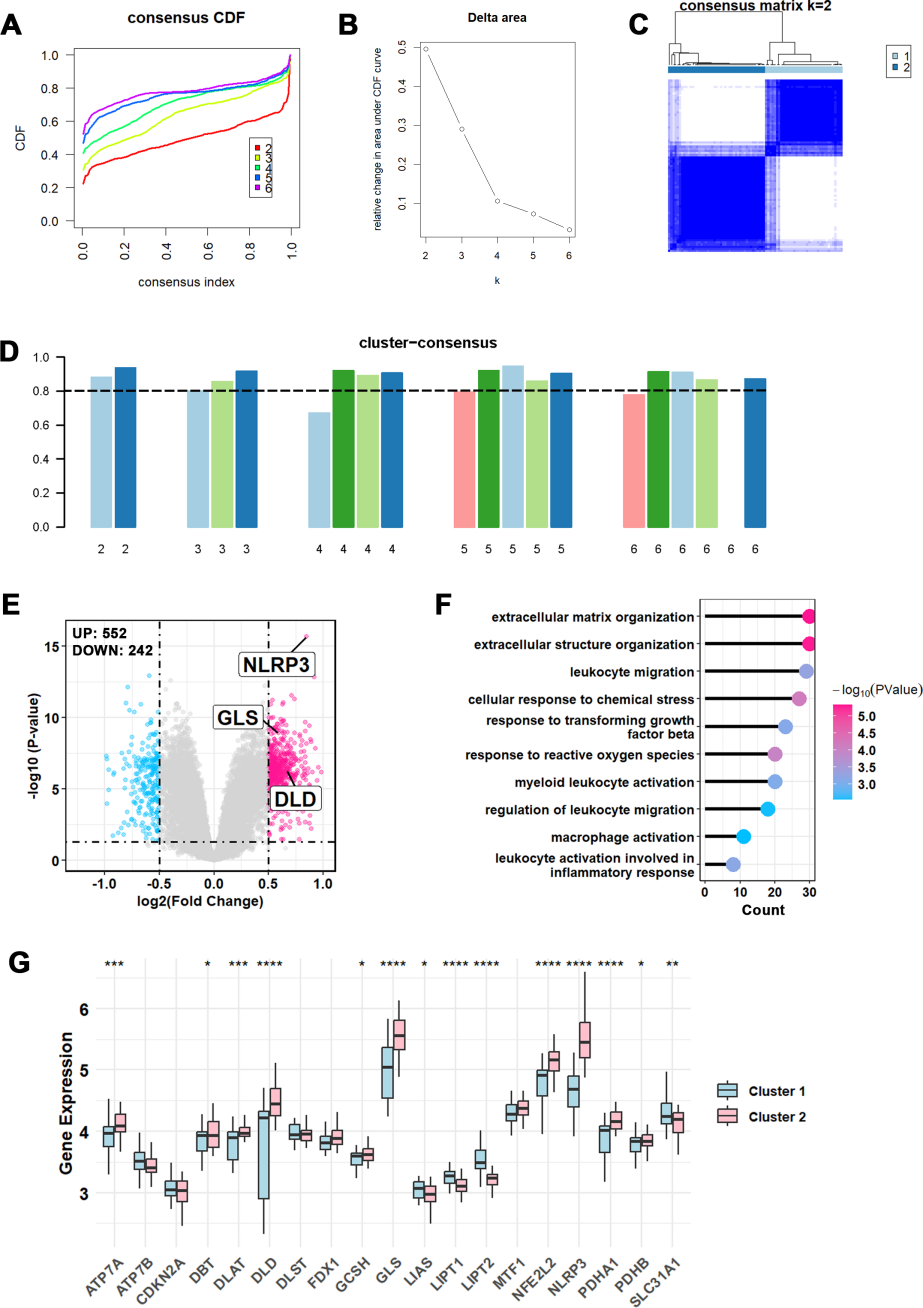
Identification of cuproptosis-related molecular subtypes and comprehensive pathway enrichment analysis. **(A)** CDF curves displayed consensus distributions from k=2 to k=6. **(B)** Area fraction of CDF curve from k=2 to k=6. **(C)** Consensus clustering matrix was generated for values of k = 2. **(D)** Cluster-consensus bar plot showing the effectiveness of clustering with 0.8 threshold. **(E)** Volcano plot showing differentially expressed genes (DEGs) of Cluster2 *vs.* Cluster1, red dots represent up-regulated DEGs and blue dots represent down-regulated DEGs. Differential CRGs are also labeled. **(F)** The lollipop graph exhibits the GO enriched pathways of Cluster2 *vs.* Cluster1. **(G)** Expression pattern of 19 CRGs in Cluster1 and Cluster2 were presented as box plot with mean ± SD. *P-values* in the box plots are denoted by asterisks: *P<0.05, **P<0.01, ***P<0.001, ****P<0.0001.

Given the association between cuproptosis and IPF, we performed consensus clustering based on CRG expression differences, revealing the presence of two robust molecular subtypes (k = 2). The cumulative distribution function (CDF) curves (**Figure 3A**) and delta area plot (**Figure 3B**) indicate that k = 2 provides the most stable clustering solution. The consensus matrix heatmap (**Figure 3C**) demonstrates clear separation between clusters with high intra-cluster consistency, and the cluster-consensus scores (**Figure 3D**) are consistently high (>0.8), confirming the reproducibility and robustness of the two-subtype classification. Moreover, the volcano plot identified 552 upregulated genes and 242 downregulated genes, with GLS, NLRP3, and DLD notably upregulated in Cluster 2, consistent with differential analysis between IPF and healthy controls (**Figure 3E**). To evaluate functional alterations across clusters with varying levels of cuproptosis involvement, enrichment analysis was performed. The results indicated that cuproptosis may impact leukocyte migration and activation, as well as extracellular matrix organization (**Figure 3F**). Further investigation of CRG expression showed that most CRGs were upregulated in Cluster 2, with GLS, NLRP3, NFE2L2, DLD, and PDHA1 being the most significant. In contrast, LIPT1 and LIPT2 were downregulated in Cluster 2 (**Figure 3G**).

Meanwhile, leave-one-gene-out analysis showed that the subtype classification remained largely stable after omission of most individual CRGs, with ARI values close to 1.0 in the majority of iterations (**Supplementary Figure 2A**). A greater impact was observed when a small number of genes, particularly NLRP3 and, to a lesser extent, GLS, were omitted, suggesting that these genes contributed more strongly to the original subtype structure.

### Hub genes associated with different subtypes of IPF

**Figure 4:**
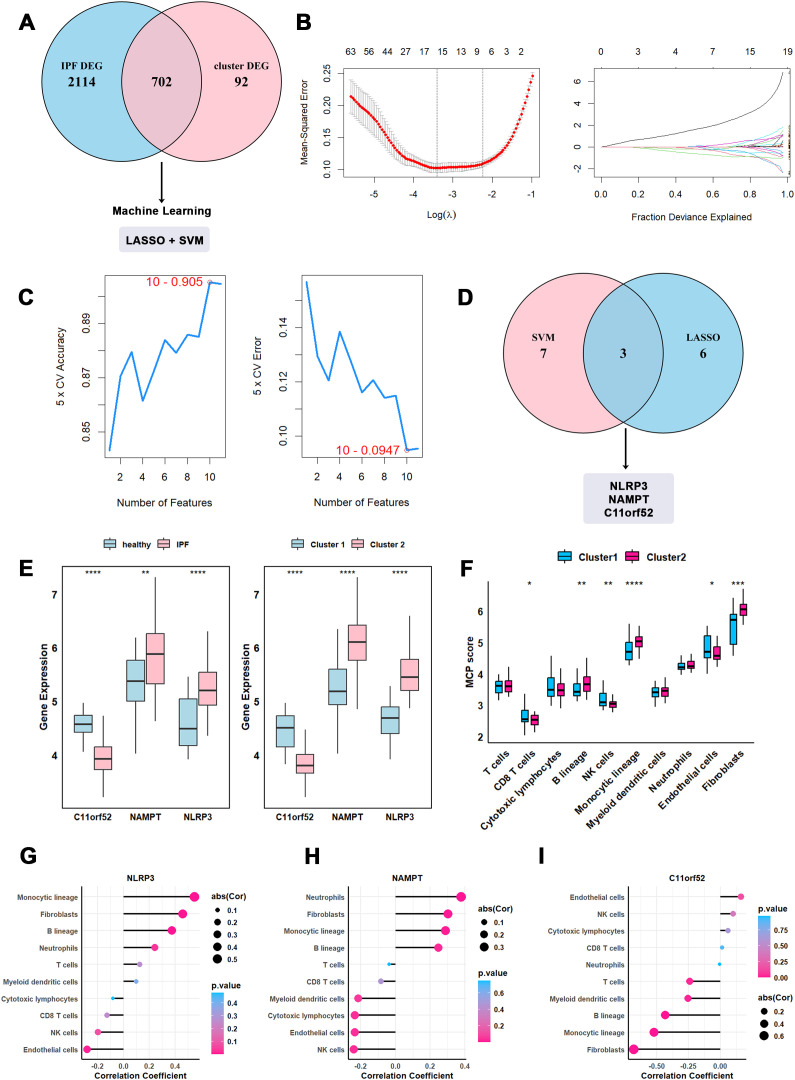
Identification of cuproptosis associated hub genes in IPF clusters. **(A)** Venn plot of DEGs of IPF *vs.* healthy control and Cluster2 *vs.* Cluster1. **(B)** Intersected DEGs profiles based on LASSO coefficients. And LASSO coefficient values of the DEGs. The vertical dashed lines of right panel are the optimal log(λ) values. **(C)** Intersected DEGs profiles based on SVM-RFE algorithm. Left panel represent 5×CV Accuracy and right panel represent 5×CV Error. **(D)** Hub genes of two algorithms overlapped as Venn diagram. **(E)** Expression trends of three hub genes of IPF *vs.* healthy control and Cluster2 *vs.* Cluster1. **(F)** MCP counter evaluate the immune infiltration level and visualized as box plot. **(G–I)** Correlation of each hub gene and immune cells and mesenchymal cells in MCP counter. Horizontal axis indicates the correlation, and the depth of dot color represent P value. *P-values* in the box plots are denoted by asterisks: *P<0.05, **P<0.01, ***P<0.001, ****P<0.0001.

We identified 702 intersecting DEGs from the comparison of Cluster 2 *vs.* Cluster 1 and IPF *vs.* healthy controls to screen for hub genes associated with IPF subtypes (**Figure 4A**). The LASSO regression and SVM-RFE algorithms were used for this screening process. LASSO regression identified 9 features, while SVM-RFE produced 10 (**Figures 4B, C**). By overlapping the feature genes from both methods, we identified three hub genes: NAMPT, NLRP3, and C11orf52 (**Figure 4D**). The expression patterns of these hub genes were visualized, showing consistent trends with NAMPT and NLRP3 upregulated, and C11orf52 downregulated in both Cluster 2 *vs.* Cluster 1 and IPF *vs.* healthy controls (**Figure 4E**). These hub genes will be investigated further in our upcoming study. Repeated resampling analyses further showed that the top candidate genes displayed varying but generally concordant selection frequencies across LASSO and SVM-RFE (**Supplementary Figure 2B**), supporting the overall stability of the hub gene selection strategy.

Given that leukocyte migration and activation were primarily enriched, we performed MCP-counter analysis to explore immune infiltration differences between Cluster 1 and Cluster 2. Monocytic lineage cells and fibroblasts showed the greatest increase in Cluster 2, suggesting that cuproptosis plays a role in the progression of IPF (**Figure 4F**). We also analyzed the correlation between the three hub genes and immune cell types identified by MCP-counter. NAMPT and NLRP3 were positively correlated with fibroblasts and monocytic lineage, while C11orf52 was negatively correlated (**Figures 4G–I**).

### Signaling pathways enrichment of hub genes

**Figure 5:**
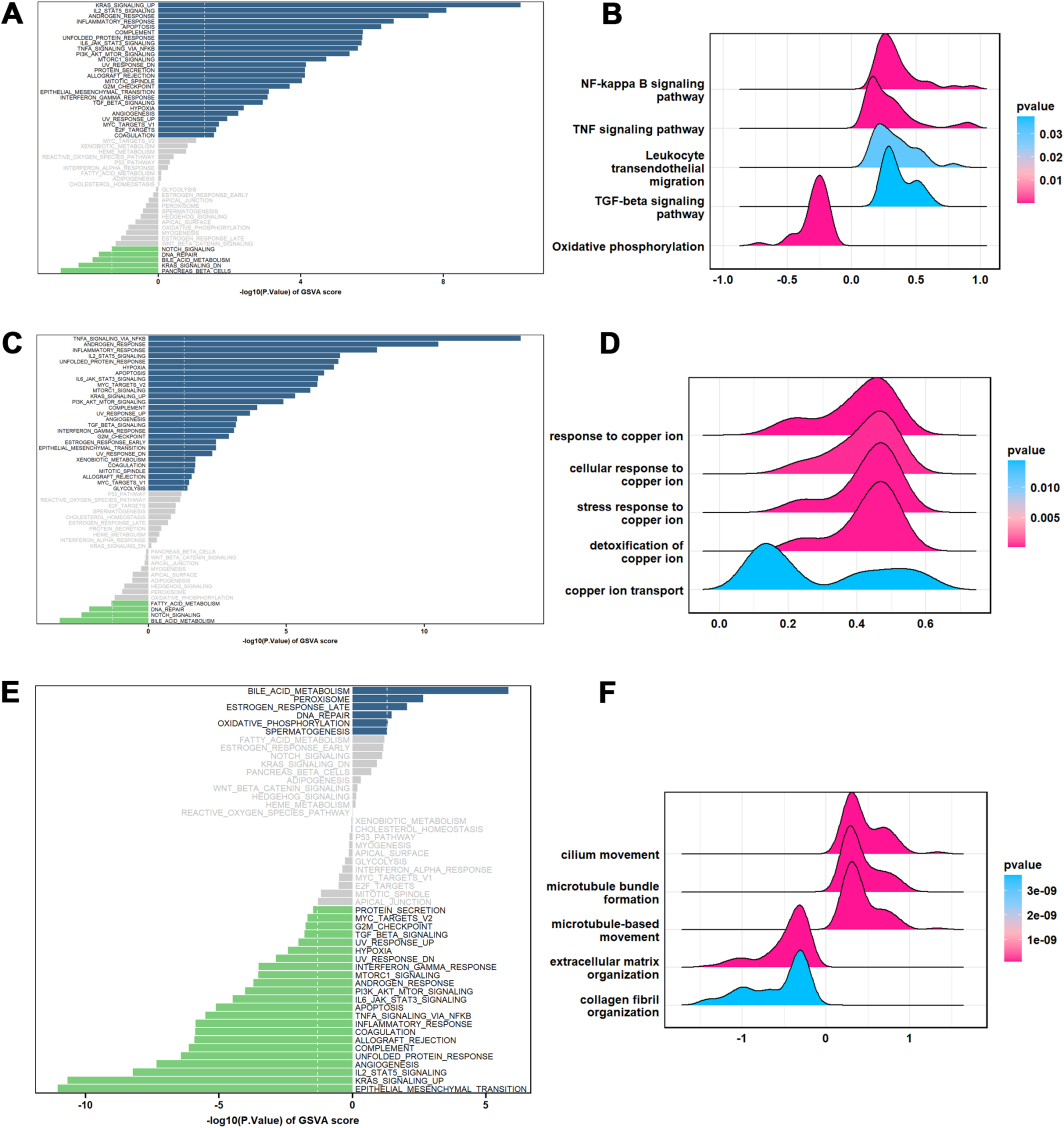
Pathway enrichment of each hub gene. **(A)** GSVA of NLRP3; **(B)** GSEA of NLRP3; **(C)** GSVA of NAMPT. **(D)** GSEA of NAMPT; **(E)** GSVA of C11orf52; **(F)** GSEA of C11orf52. Differences in pathway activities scored by GSVA, between high expression and low expression. The GSVA scores, serving as the X-axis, are sorted in descending order according to their rank, to reflect the significant pathways enrichment levels of gene set, and two K-S statistical distribution lines are drawn. Blue and green colors represent significantly enriched pathways, while gray color indicates non-differential pathways. GSEA, Gene Set Enrichment Analysis; NES, Normalized Enrichment Score.

To explore the molecular mechanisms by which these three hub genes influence IPF progression, we performed pathway enrichment analysis using GSVA and GSEA. High NLRP3 expression was primarily associated with the activation of pathways such as KRAS signaling, inflammatory response, IL2-STAT5, and apoptosis (**Figure 5A**). Additionally, NLRP3 activated pathways related to NF-kappa B, TNF, and TGF-β signaling while downregulating oxidative phosphorylation (**Figure 5B**). Similarly, NAMPT also activated inflammatory pathways, consistent with the behavior of NLRP3 (**Figure 5C**). Interestingly, GSEA revealed that NAMPT was highly enriched in cellular copper homeostasis pathways, including stress responses to copper ions and copper ion transport, suggesting that NAMPT may serve as a potential cuproptosis-regulating gene in IPF (**Figure 5D**). In contrast, C11orf52 displayed anti-inflammatory and anti-fibrotic effects, as evidenced by the downregulation of epithelial-mesenchymal transition, inflammatory response, and TGF-β signaling (**Figure 5E**). Phenotypically, high expression of C11orf52 was associated with enhanced cilium and microtubule-based movement, as well as reduced extracellular matrix and collagen fibril organization (**Figure 5F**).

### External validation of hub genes

**Figure 6:**
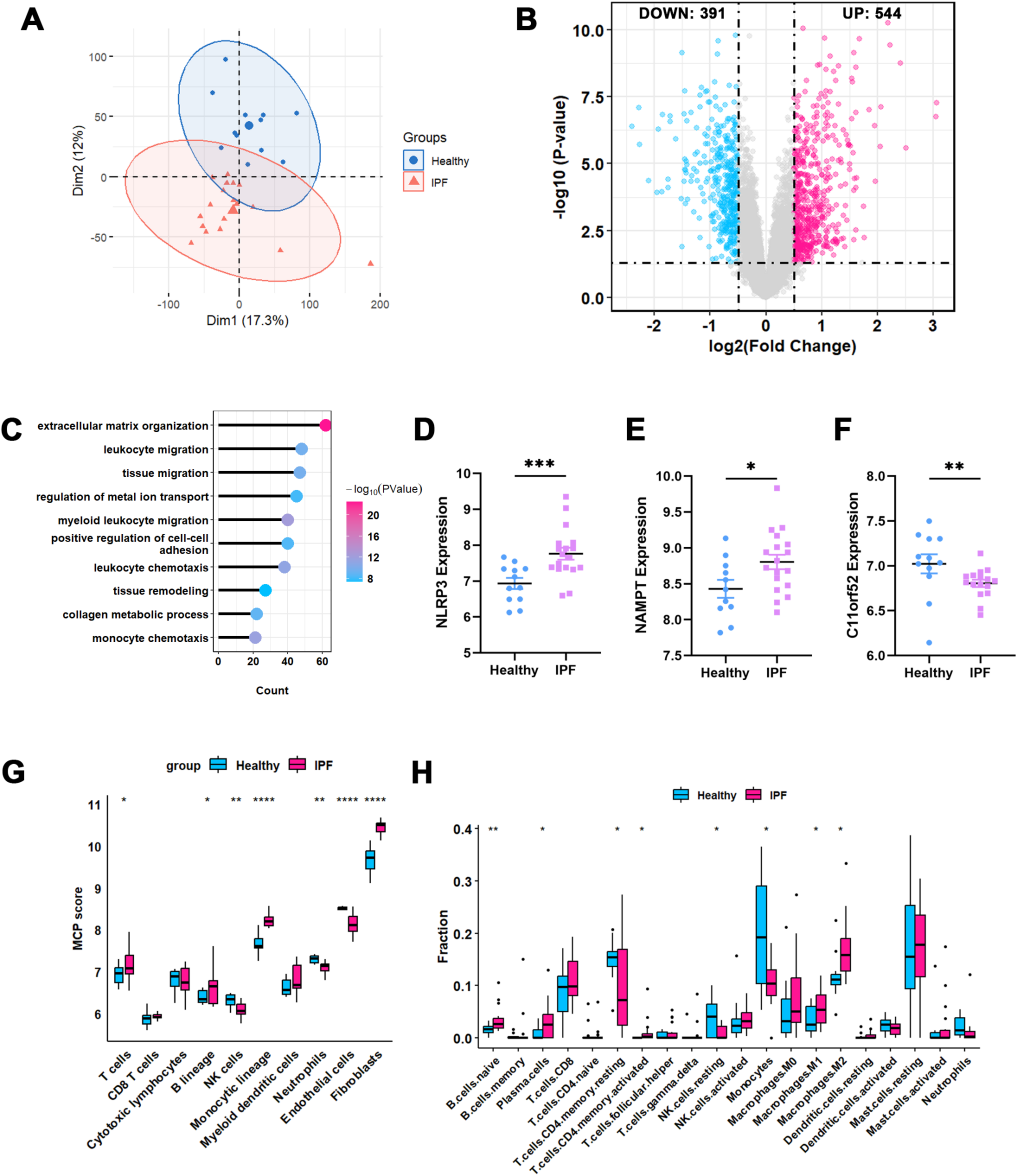
Validation of hub genes using external bulk RNA-seq datasets. **(A)** PCA demonstrates the comparability between the integrated bulk RNA-seq datasets. **(B)** Volcano plot illustrating the DEGs in IPF compared to healthy controls. **(C)** Lollipop plot displaying the GO enrichment analysis of DEGs between IPF and healthy controls. **(D–F)** Expression profiles of the three hub genes in IPF versus healthy controls, validating their differential expression trends. **(G)** Immune cell infiltration in IPF and healthy controls, assessed using CIBERSORT, visualized through box plots. **(H)** MCP counter analysis of immune infiltration levels in IPF and healthy controls, also represented as box plots. *:<0.05; **: <0.01; ***: <0.001; ****: <0.0001.

To validate the identified hub genes, we used the GEO datasets GSE72073 and GSE48149, which include 18 IPF samples and 12 healthy controls. The “Combat” function was applied to integrate these two datasets, ensuring their comparability and accounting for heterogeneity (**Figure 6A**). We identified 544 upregulated and 391 downregulated genes, enriched in Gene Ontology (GO) terms related to immune regulation and tissue remodeling (**Figures 6B, C**). Consistent with our primary analysis (**Figure 4E**), NLRP3 and NAMPT were upregulated in IPF samples, while C11orf52 was upregulated in healthy controls (**Figures 6D–F**). Regarding immune infiltration, MCP-counter analysis showed significant infiltration of fibroblasts and monocytic lineage cells in IPF samples (**Figure 6G**). Additionally, CIBERSORT analysis indicated that M1 and M2 macrophages were the dominant monocytic lineage cells infiltrating IPF tissues (**Figure 6H**). It is important to note that validation in these external datasets focused on differential expression and immune infiltration, while higher-order analyses remain exploratory in the discovery datasets.

### Validation of hub genes at the single cell level

**Figure 7:**
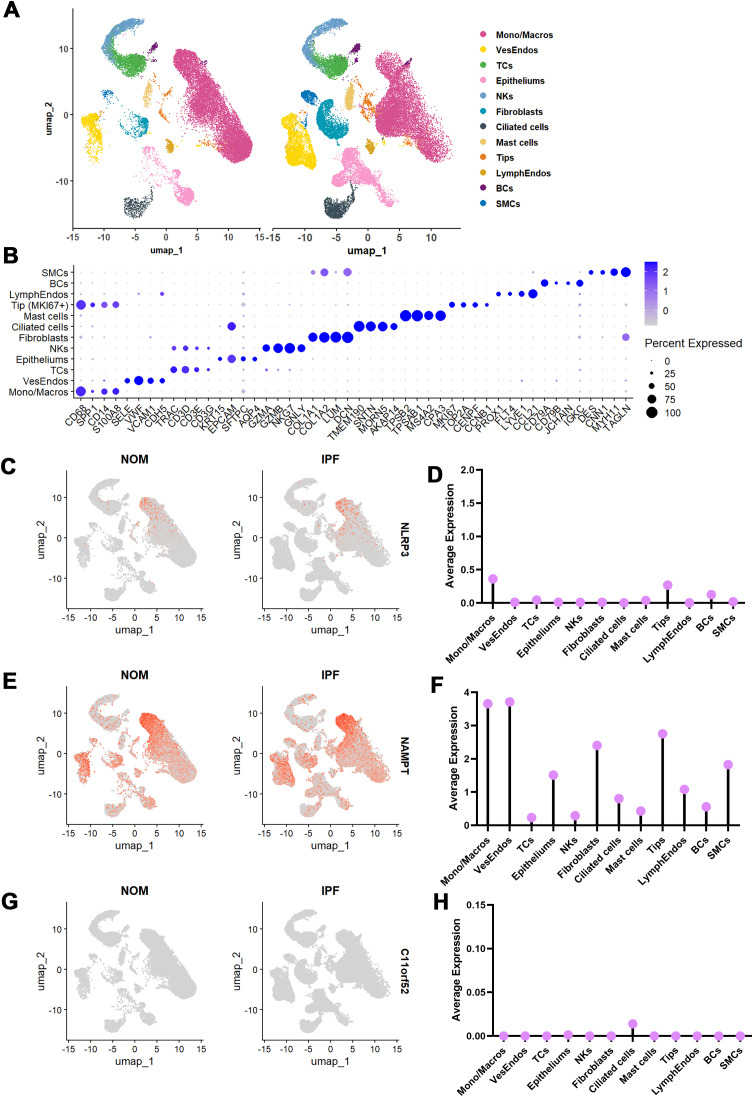
Single-cell landscape of hub genes. **(A)** UMAP plots depict 12 major cell types as identified by clustering, with each dot representing an individual cell color-coded by type. **(B)** Dot plot represents the cell markers employed in cell type identification. **(C, D)** Feature plot and average expression of NLRP3 in each cell type. **(E, F)** Feature plot and average expression of NAMPT in each cell type. **(G, H)** Feature plot and average expression of C11orf52 in each cell type.

A total of 16 samples (eight normal and eight IPF samples) were analyzed using the scRNA-seq dataset (GSE128033). Of these, 20,893 single cells were derived from normal tissues, while 28,973 cells were from IPF samples. The cells were classified into 12 main cell types, with each type well-distributed across the samples (**Figure 7A**). Based on established markers, each cell type was accurately labeled (**Figure 7B**). To validate the expression levels of the hub genes, we calculated the average expression for each gene in the IPF cell types. NLRP3, reported frequently in recent studies, was moderately expressed in monocytes/macrophages and tip cells, which may involve in angiogenesis (**Figures 7C, D**). NAMPT, a key enzyme implicated in various pathological processes including metabolic regulation, inflammatory response, and protein secretion, was highly expressed in mesenchymal cells and immune cells, particularly in monocytes/macrophages (**Figures 7E, F**). This suggests that NAMPT may play a role in the crosstalk between mesenchymal cells and monocytes/macrophages. In contrast, C11orf52 showed limited expression, primarily in ciliated cells (**Figures 7G, H**). As a result, NAMPT became the central focus of further investigation in this study.

### NAMPT was positively correlated with cuproptosis in fibroblasts

**Figure 8:**
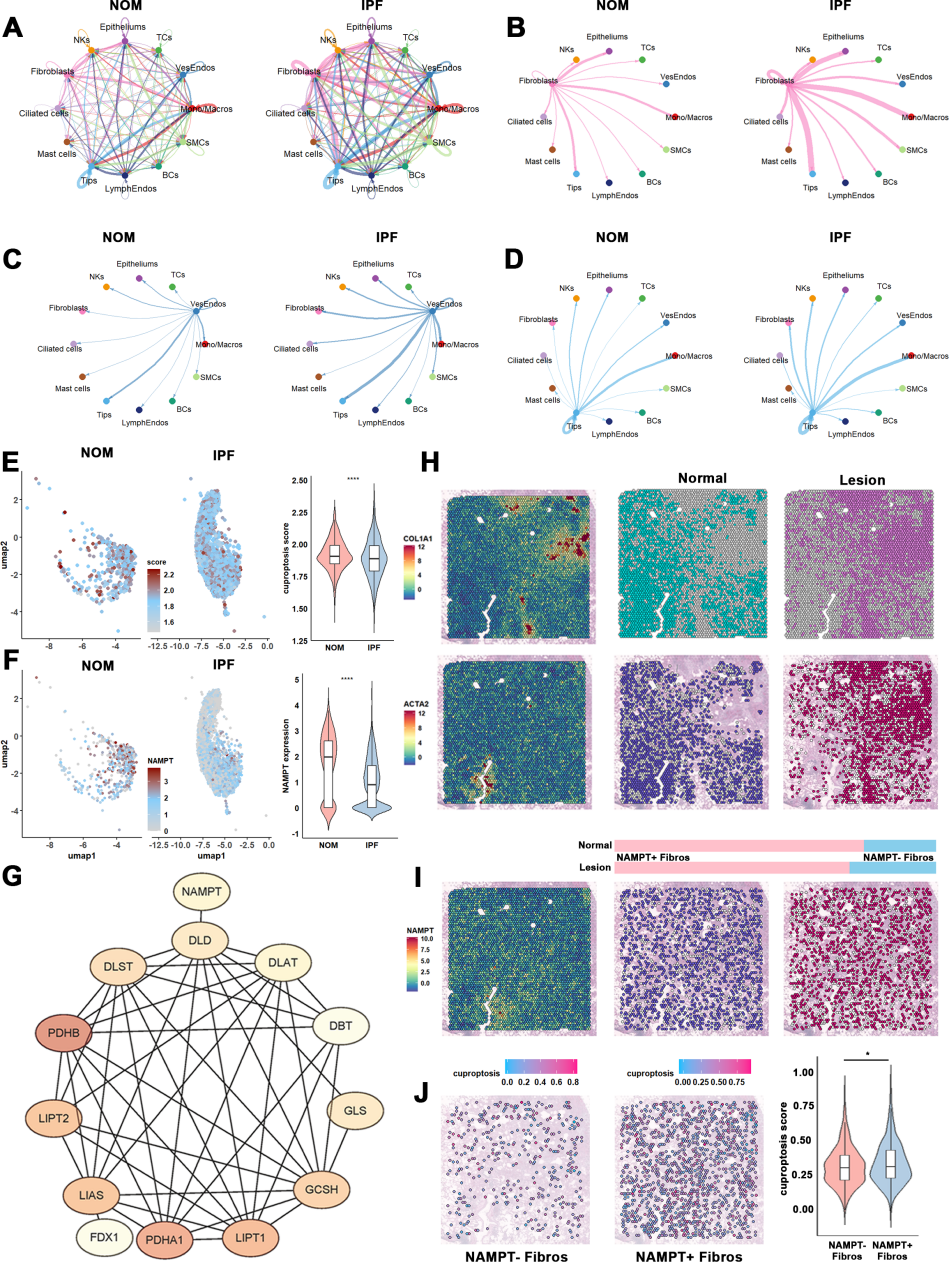
Cell-cell communication and spatial transcriptome identify the regulatory correlation of NAMPT and cuproptosis in IPF. **(A)** Circle plots map the interaction numbers among the 12 major cell subclusters in control and IPF group. **(B–D)** Circle plots detail the interaction numbers among fibroblasts, vascular endothelial cells, Tip cells and Mono/Macros in IPF and healthy control. **(E)** Cuproptosis GSVA score were represented as UMAP plot and violin plot, depth of dot colors indicates the value of the score. **(F)** NAMPT expression were represented as UMAP plot and violin plot, depth of dot colors indicates the expression level. **(G)** PPI network exhibits the interaction of NAMPT and cuproptosis. **(H)** Upper panel represent the expression of COL1A1, dividing the normal (blue) and lesion (red) area. Lower panel exhibit the expression of ACTA2, and visualize fibroblasts spot in normal (deep blue) and lesion area (deep red). **(I)** NAMPT expression were established, NAMPT positive fibroblasts in normal (deep blue) and lesion (deep red) area. Bar plot exhibit the proportion of NAMPT positive fibroblasts in normal and lesion area. **(J)** Cuproptosis GSVA score were represented in lesion area NAMPT positive and negative fibroblasts, and quantified in violin plot. *P-values* in the box plots are denoted by asterisks: *P<0.05.

In **Figure 7F**, NAMPT was predominantly expressed in vascular endothelial cells, tip cells, fibroblasts, and monocytes/macrophages. Based on this, we utilized CellChat to identify significant changes in the crosstalk between fibroblasts and monocytes/macrophages in IPF (**Figures 8A–D**). Next, GSVA was applied to compare control fibroblasts and IPF fibroblasts, revealing a marked decrease in cuproptosis involvement in IPF fibroblasts (**Figure 8E**). We also visualized changes in NAMPT expression in fibroblasts, observing a significant downward trend (**Figure 8F**). As depicted in **Figure 8G**, NAMPT may play a regulatory role in cuproptosis.

To further validate the relationship between NAMPT and cuproptosis, we employed spatial transcriptomics. In the IPF sample, we identified 4,882 spots, classifying 2,391 as lesion spots and 2,491 as normal spots based on collagen deposition levels. Fibroblast-enriched spots were identified using the ACTA2 marker (**Figure 8H**). We then categorized the fibroblast-containing spots into NAMPT+ and NAMPT- spots within both lesion and normal areas. The NAMPT+ spots were significantly reduced in lesion areas, consistent with our single-cell sequencing results (**Figure 8I**). Finally, GSVA performed on fibroblast spots from the lesion areas showed that NAMPT+ fibroblasts exhibited higher levels of cuproptosis, suggesting that NAMPT may facilitate the regulation of cuproptosis in pathogenic fibroblasts within IPF (**Figure 8J**).

**Figure 9:**
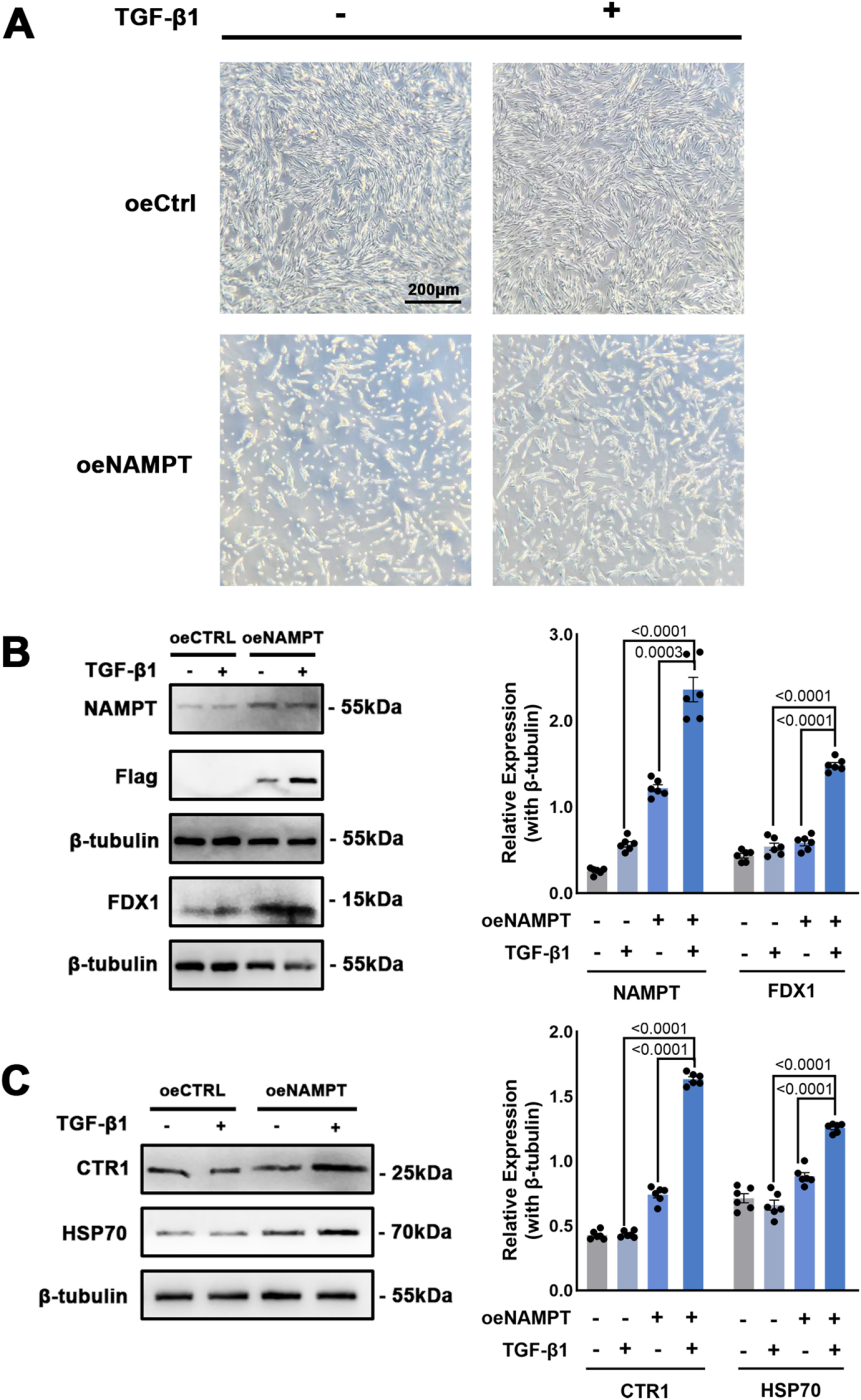
Overexpression of NAMPT promotes the expression of cuproptosis-related proteins in MRC-5 cells. **(A)** Phase-contrast images of MRC5 fibroblasts transfected with control vector (oeCtrl) or NAMPT overexpression plasmid (oeNAMPT), treated with or without TGF-β1 (10 ng/mL) for 48 h. Scale bar = 200 μm. **(B)** Western blot detection of NAMPT and FDX1 levels in the MRC-5 cells, and flag tag demonstrates the overexpression of NAMPT. **(C)** Expression level of CTR1 and HSP70 indicated cuproptosis occurrence in human lung fibroblasts. Mean ± SEM with p value indicated in the image, unpaired *t*-test.

To further investigate the pro-cuproptosis role of NAMPT in fibroblasts, we utilized MRC-5 cells, a widely used human fibroblast cell line. NAMPT overexpression plasmids were transfected into MRC-5 cells, and transforming growth factor-β1 (TGF-β1) was applied to simulate fibrotic progression. Notably, significant cell death was observed under the microscope 48 hours post-transfection (**Figure 9A**). To confirm the induction of cuproptosis, Western blot analysis was performed. NAMPT expression was markedly elevated following plasmid transfection and further enhanced by TGF-β1 treatment. FDX1, a classical marker of cuproptosis, was also upregulated under these conditions (**Figure 9B**). Additionally, two other cuproptosis-associated proteins, CTR1 and HSP70, exhibited similar expression trends, further supporting our hypothesis (**Figure 9C**).

### Potential cuproptosis mechanisms of IPF regulated by NAMPT through crosstalk between M2 macrophages and fibroblasts (SFRP1+)

We next sought to determine whether the increased expression of NAMPT in fibroblasts was associated with altered intercellular communication in IPF. To address this, we used CellChat (24), a tool that leverages a database of ligand-receptor interactions to analyze cell-cell communication from scRNA-seq data. These interactions were inferred computationally, highlighting potential ligand-receptor communications that may contribute to fibroblast-macrophage crosstalk. Our analysis revealed significant differences in cell-cell interactions between control and IPF samples (**Figure 8A**). Notably, we observed an apparent increase in the predicted interaction strength between fibroblasts and various immune cells, particularly monocytes/macrophages. This finding aligns with our previous results from the bulk RNA-seq dataset (**Figure 4F**), further supporting the notion of potentially enhanced communication between fibroblasts and immune cells in IPF.

**Figure 10:**
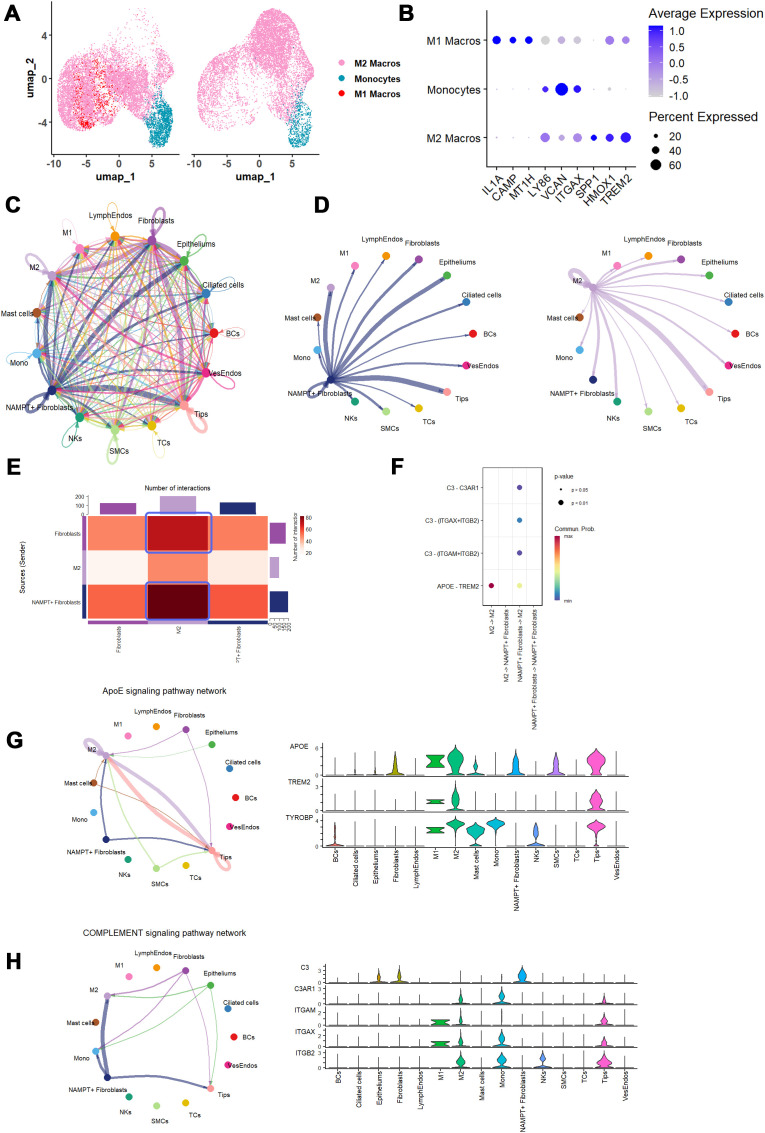
Cell-cell communication dynamics between mono/macros and fibroblasts expressing identified NAMPT in IPFs. **(A)** UMAP plots major Mono/Macros types as identified by clustering. **(B)** Dot plot represents the cell markers employed in cell type identification. **(C)** Circle plots map the interaction numbers among the cell subclusters in IPF group. **(D)** Circle plots detail the interaction numbers between fibroblasts expressing NAMPT, and other cell subclusters in IPFs, with a focus on M2 macrophages interactions. **(E)** Heatmap explore the interaction numbers between NAMPT positive fibroblasts and M2 macrophages, differentiated by NAMPT expression levels, with color gradients reflecting scaled interaction numbers. **(F)** A dot plot highlights the primary signaling pathways between NAMPT positive fibroblasts and M2 macrophages in IPFs, where each dot represents a ligand-receptor pair, sized by pathway involvement *P-value*, and colored by communication probability. **(G, H)** An illustration of ApoE and COMPLEMENT signaling pathways, including a circle plot (left) showing interaction numbers and a violin plot (right) depicting ligand and receptor gene expression levels.

We further subdivided monocytes/macrophages into three subclusters—monocytes, M1, and M2—to explore their interactions with high NAMPT-expressing fibroblasts in IPF samples (**Figures 10A, B**). Interestingly, our findings revealed that NAMPT+ fibroblasts were predicted to act as primary signal senders in crosstalk with M2 macrophages, contrasting with the conventional view that M2 macrophages are the main signal senders (**Figures 10C, D**). Additionally, the number of interactions between NAMPT+ fibroblasts and M2 macrophages was stronger than those involving NAMPT- fibroblasts, suggesting that NAMPT+ fibroblasts may potentially have a stronger interaction with M2 macrophages in IPF (**Figure 10E**).

Further analysis of the interaction mechanisms among NAMPT+ fibroblasts, NAMPT- fibroblasts, and M2 macrophages revealed that NAMPT+ fibroblasts predominantly engage with M2 macrophages via ApoE signaling (ApoE - TREM2) and the COMPLEMENT pathway (C3 - (C3AR1/ITGAX + ITGB2/ITGAM + ITGB2)) (**Figures 9F**, **10G, H**, left panel, **Supplementary Figure 3**). We also observed increased expression of the corresponding receptors and ligands for these pathways in both NAMPT+ fibroblasts and M2 macrophages, consistent with the predicted interactions (**Figures 10G, H**, right panel).

**Figure 11:**
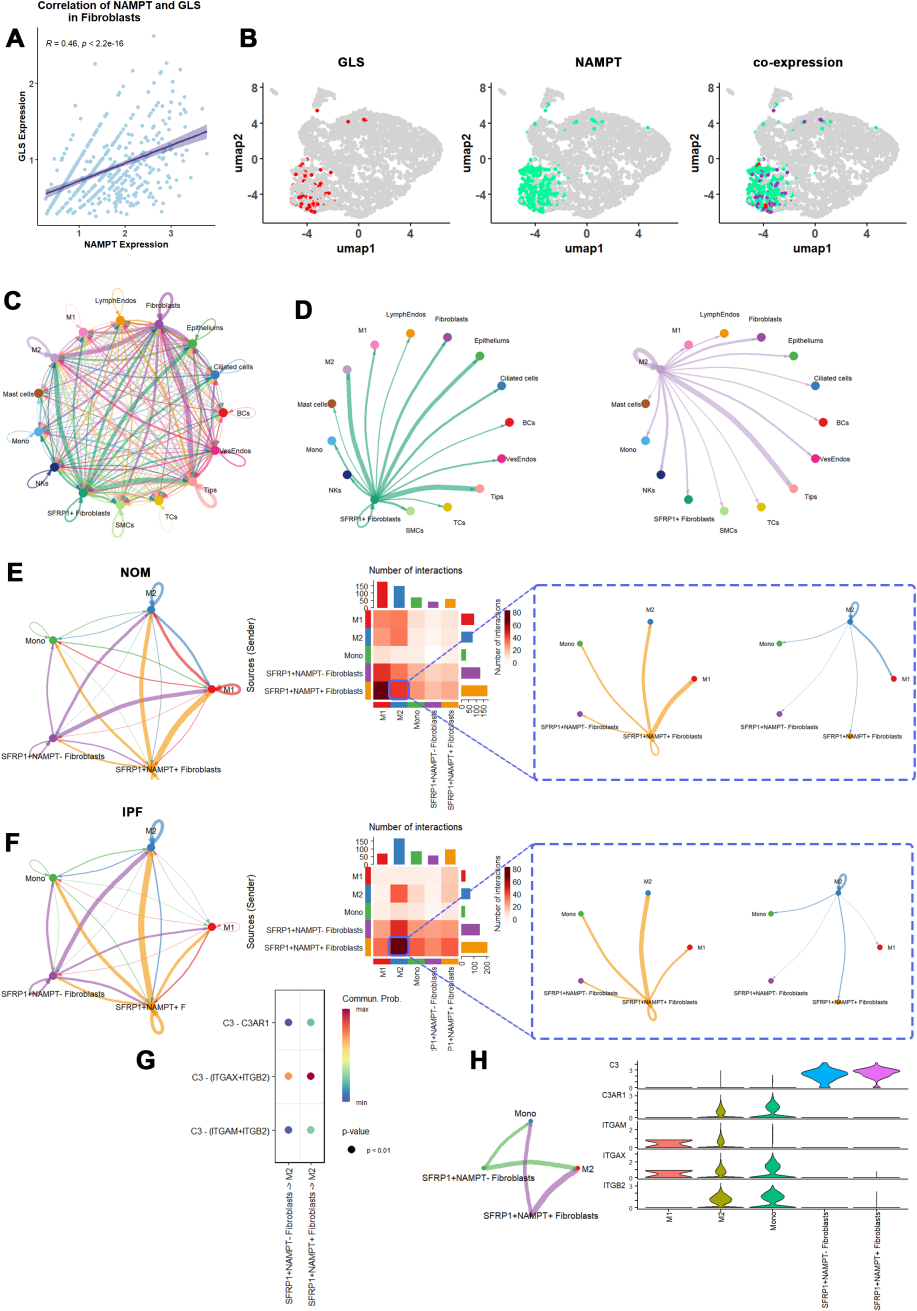
NAMPT expression in SFRP1+ fibroblasts is suggested to enhance fibroblasts-M2 macrophages interaction in IPFs. **(A)** A scatter plot shows the correlation of NAMPT and GLS expression in fibroblasts. **(B)** Co-expression of NAMPT and GLS in SFRP1+ fibroblasts were visualized as UMAP, and each dot represents a single cell. **(C)** Circle plots assess the interaction numbers between SFRP1+ fibroblasts and other cell type. **(D)** Circle plots detail the interaction numbers between SFRP1+ fibroblasts expressing NAMPT, and other cell subclusters in IPFs. **(E)** Circle plots and heatmap explore the interaction numbers between NAMPT+SFRP1+ fibroblasts and M2 macrophages, differentiated by NAMPT expression levels, with color gradients reflecting scaled interaction numbers in healthy control. **(F)** Circle plots and heatmap explore the interaction numbers between NAMPT+SFRP1+ fibroblasts and M2 macrophages, differentiated by NAMPT expression levels, with color gradients reflecting scaled interaction numbers in IPFs. **(G)** A dot plot highlights the primary signaling pathways between NAMPT+SFRP1+ fibroblasts and M2 macrophages in IPFs, where each dot represents a ligand-receptor pair, sized by pathway involvement *P-value*, and colored by communication probability. **(H)** An illustration of COMPLEMENT signaling pathways, including a circle plot (left) showing interaction numbers and a violin plot (right) depicting ligand and receptor gene expression levels.

Given the phenotypic changes induced by NAMPT expression in fibroblasts (**Figure 9D**), we aimed to further explore and validate the correlation between NAMPT and cuproptosis in specific fibroblast subclusters. Consistent with bulk RNA-seq data, NAMPT expression in fibroblasts showed a positive correlation with GLS, a key cuproptosis marker. (**Figure 11A**). The analysis revealed a significant positive correlation between NAMPT and GLS in fibroblasts. Additionally, colocalization analysis indicated that NAMPT and GLS were co-expressed in the SFRP1+ fibroblast population (**Figure 11B**; **Supplementary Table S1** listed the marker genes of fibroblast subcluster). These findings indicate a potential relationship between NAMPT and cuproptosis at single-cell resolution.

We proceeded by isolating SFRP1+ fibroblasts from IPF samples to assess their interactions with other cell clusters using CellChat analysis. Predicted interactions indicated that SFRP1+ fibroblasts may serve as primary signal transducers with M2 macrophages (**Figures 11C, D**).

Lastly, we analyzed the ligand-receptor pathways between SFRP1+ fibroblasts and M2 macrophages with either positive or negative NAMPT expression. Our results showed that interactions between SFRP1+NAMPT+ fibroblasts and M2 macrophages were significantly enhanced in IPF samples (**Figures 11E, F**). The most notable change was observed in the COMPLEMENT pathway, highlighting potential involvement in microenvironmental signaling (**Figure 11G**; **Supplementary Figure 4**). Specifically, the key ligand-receptor pair involved in this pathway was C3-ITGB2 (**Figure 11H**). These findings suggest that SFRP1+ fibroblasts may influence the progression of IPF through ligand-receptor interactions with M2 macrophages. This, in turn, may enhance M2 macrophages’ role in shaping a pro-fibrotic microenvironment, driving the progression of IPF.

**Figure 12:**
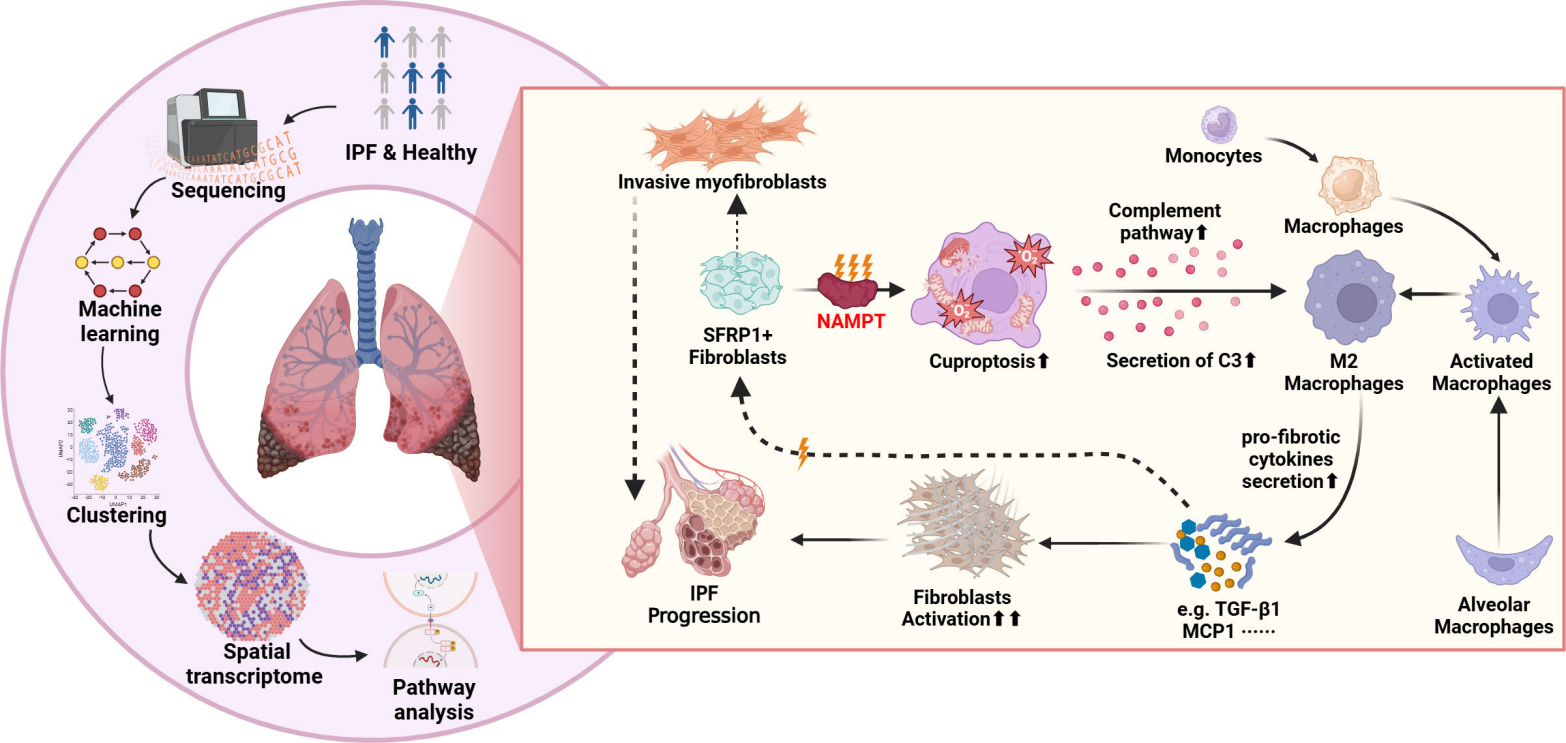
Cell-to-cell communication between SFRP1^+^ fibroblasts and M2 macrophages in the IPF microenvironment. Cuproptosis of SFRP1^+^ fibroblasts enhances their interaction with M2 macrophages. In response, M2 macrophages secrete pro-fibrotic factors, including TGF-β1, MCP-1, and collagens, promoting remodeling of the fibrotic microenvironment in idiopathic pulmonary fibrosis patients.

## Discussion

Copper ions are responsible for collagen cross-link, and extracellular matrix (ECM) remodeling, which is central processes in the progression of fibrotic diseases (25, 26). Excessive deposition of ECM components ultimately leads to irreversible tissue scarring and loss of function. Dysregulation of copper homeostasis has been linked to enhanced fibrogenesis, as elevated copper levels are associated with increased activity in fibrotic tissues (27). Therefore, investigating copper-dependent cell death pathways, such as cuproptosis, provides an additional layer for understanding fibrotic progression.

Through transcriptomic analysis and functional validation, we identified NAMPT as a key regulator in fibroblasts-associated pathogenic processes in IPF. NAMPT, a critical enzyme in NAD+ biosynthesis and cellular metabolism, has been implicated in multiple pathological conditions, including aging, metabolic disorders and cardiomyopathy (28–30). Previous studies have also demonstrated that NAD^+^ metabolism can regulate immune invasion and cellular plasticity (31). In our study, NAMPT plays a particularly important role in fibroblast biology, influencing cellular metabolism and functional states that are closely linked to aberrant tissue remodeling in IPF.

Our results revealed an overall increase in cuproptosis-related signatures in IPF at the bulk tissue level, suggesting activation of copper-dependent stress responses within the fibrotic microenvironment. However, fibroblast-specific analysis showed a relative decrease in cuproptosis involvement, which initially appears contradictory. Rather than representing a technical limitation, this difference likely reflects cell-type- and state-specific regulation within the lung microenvironment. The elevated cuproptosis signals observed in bulk RNA-seq may arise from multiple cell populations under stress conditions, whereas fibroblast-specific analysis captures intrinsic adaptive responses that promote cell survival.

It is important to emphasize that, although the same gene set and GSVA parameters were applied across bulk RNA-seq, single-cell RNA-seq, and spatial transcriptomic datasets, these platforms differ substantially in normalization framework, data sparsity, and biological resolution. Bulk RNA-seq primarily reflects tissue-level average signals, single-cell RNA-seq captures cell-type-specific transcriptional states at higher resolution, and spatial transcriptomics represents spot-level signals influenced by mixed-cell composition. Accordingly, GSVA results obtained from each platform were interpreted within their respective analytical contexts.

Importantly, fibroblasts in IPF exhibit substantial heterogeneity. Previous studies have identified SFRP1+ fibroblasts as a transitional and relatively less invasive state that precedes the emergence of pathogenic myofibroblasts (32). Consistent with this concept, our data further revealed functional heterogeneity within fibroblasts, where a NAMPT+ subset displays higher cuproptosis-related signatures and enhanced intercellular communication with M2 macrophages. These findings suggest that cuproptosis-related activity in fibroblasts may not simply reflect cell death, but rather a distinct metabolic and signaling state.

Based on these observations, we propose that NAMPT-associated cuproptosis in SFRP1+ transitional fibroblasts may be linked to altered intercellular communication, particularly through pathways such as COMPLEMENT signaling. This state may enhance fibroblast–macrophage crosstalk, contributing to microenvironmental remodeling and promoting fibrotic progression. In this context, cuproptosis may function not only as a cell death mechanism but also as a regulator of fibroblast behavior and signaling capacity.

At the same time, the overall reduction of cuproptosis signatures in fibroblasts may indicate a shift toward more persistent and stress-resistant fibroblast populations during IPF progression. Loss or functional alteration of transitional fibroblast states, such as SFRP1+ cells, may reduce constraints on fibroblast activation, thereby favoring the accumulation of more profibrotic fibroblast phenotypes. This interpretation consistent with mechanisms of resistance to programmed cell death in fibrotic diseases (33, 34).

Align with our results, previous studies have shown that complement signaling contributes to lung fibrosis progression and is associated with poor prognosis (35, 36). Our analysis suggests that NAMPT-associated cuproptosis may enhance fibroblast–macrophage interactions via the C3–ITGB2 complement pathway. Although the direct regulatory link between cuproptosis and complement activation remains to be fully elucidated, cellular stress and metabolic alterations are known to modulate complement signaling (37). Fibroblasts undergoing cuproptosis-associated stress may release signals that activate complement pathways, thereby promoting M2 macrophage polarization and reinforcing pro-fibrotic signaling networks.

M2 macrophages, often referred to as “wound-healing macrophages,” play a central role in fibrosis by secreting pro-fibrotic cytokines such as TGF-β, which drive fibroblast activation and differentiation into myofibroblasts. The reciprocal interaction between fibroblasts and macrophages forms a self-sustaining loop that promotes continuous tissue remodeling (38, 39). Our findings suggest that NAMPT may contribute to this process by simultaneously modulating fibroblast metabolic state and enhancing pro-fibrotic intercellular communication.

Importantly, NAMPT-mediated cuproptosis may represent a context-dependent regulatory mechanism. Under physiological conditions, increased sensitivity to copper-induced cell death may serve as a feedback mechanism to limit excessive fibroblast proliferation. However, in IPF, this regulatory axis may be dysregulated, leading to altered fibroblast survival and enhanced pro-fibrotic signaling. This concept aligns with the broader understanding of fibrosis as a maladaptive wound-healing response, in which regulatory mechanisms are hijacked to sustain disease progression (40, 41).

From a therapeutic perspective, these findings suggest that targeting NAMPT may represent a novel strategy to modulate fibroblast behavior and disrupt fibrotic progression. Inhibition of NAMPT could potentially reduce fibroblast metabolic activity and attenuate fibroblast–macrophage crosstalk, thereby limiting ECM deposition and tissue remodeling. Given the complex interplay between fibroblasts, immune cells, and ECM, targeting metabolic regulators such as NAMPT may provide new opportunities for intervention in IPF (42).

## Conclusion

This study identifies NAMPT as a key regulator of cuproptosis in IPF fibroblasts and highlights its role in shaping the fibrotic microenvironment through interactions with M2 macrophages. These findings provide new insights into the metabolic regulation of fibroblast behavior and its contribution to IPF progression, and suggest that NAMPT may represent a potential therapeutic target for modulating fibroblast activation and copper-dependent cell death.

Several limitations should be acknowledged. Although ComBat was applied to normalized data to reduce batch effects, residual biases cannot be completely excluded. In addition, the main bioinformatic findings are primarily derived from discovery datasets, with external validation limited to hub gene expression. The identification of molecular subtypes and hub genes also depends on the selected gene sets and analytical strategies, and further validation in independent cohorts would help to strengthen these findings. Variability across transcriptomic platforms, together with the use of computational inference for cell–cell interactions, may also introduce a degree of uncertainty, and GSVA results should be interpreted in the context of the specific data characteristics of each transcriptomic platform. Furthermore, future studies incorporating non-fibrotic DPLD cohorts may provide additional context for interpreting these findings.

Despite these limitations, our integrative multi-omics analysis provides a coherent framework linking NAMPT-associated cuproptosis to fibroblast heterogeneity and immune microenvironment remodeling in IPF. These findings offer a foundation for future mechanistic studies and may facilitate the development of targeted therapeutic strategies.

## Data Availability

The bulk IPF dataset analyzed in the present study can be reviewed at the Gene Expression Omnibus (GEO: GSE53845, GSE110147, GSE72073, GSE48149). The single-cell transcriptome dataset of IPF patients are available at https://www.ncbi.nlm.nih.gov/geo/query/acc.cgi?acc=GSE128033. The spatial transcriptomics dataset was downloaded from the BioStudies repository (https://www.ebi.ac.uk/biostudies/studies/) with the accession number S-BSST1410.

## Glossary

ACTA2: Actin alpha 2
ApoE: Apolipoprotein E
C11orf52: Chromosome 11 open reading frame 52
C3: Complement component 3
C3AR1: Complement component 3a receptor 1
CAMP: Cathelicidin antimicrobial peptide
CDF: Cumulative distribution function
CIBERSORT: Cell-type Identification by estimating relative subsets of RNA transcripts
COL1A1: Collagen type I alpha 1 chain
CRGs: Cuproptosis-related genes
CTR1/SLC31A1: Copper Transport Protein 1/Solute carrier family 31, member 1
DEGs: Differentially expressed genes
DLD: Dihydrolipoamide dehydrogenase
ECM: Extracellular matrix
FDX1: Ferredoxin 1
GEO: Gene Expression Omnibus
GLS: Glutaminase
GSEA: Gene set enrichment analysis
GSVA: Gene set variation analysis
HMOX1: Heme oxygenase 1
HSP70: Heat shock protein 70
IL1A: Interleukin 1 alpha
IL2: Interleukin 2
IPF: Idiopathic pulmonary fibrosis
ITGAM: Integrin subunit alpha M
ITGAX: Integrin subunit alpha X
ITGB2: Integrin beta-2
LASSO: Least absolute shrinkage and selection operator
LIMMA: Linear Models for Microarray Data
LIPT1: Lipoyltransferase 1
LIPT2: Lipoyltransferase 2
LY86: Lymphocyte antigen 86
Mono/Macros: Monocytes/Macrophages
MsigDB: Molecular signatures database
MT1H: Metallothionein 1H
NAD: Nicotinamide adenine dinucleotide
NAMPT: Nicotinamide phosphoribosyltransferase
NFE2L2: Nuclear factor, erythroid 2 like 2
NF-κB: Nuclear factor kappa-B
NLRP3: Nucleotide- binding oligomerization domain, leucine- rich repeat and pyrin domain- containing 3
PCA: Principal component analysis
PDHA1: Pyruvate dehydrogenase E1 subunit alpha 1
PD-L1: Programmed cell death-ligand 1
PERMANOVA: Permutational multivariate analysis of variance
RNA-seq: Bulk RNA sequencing
scRNA-seq: Single-cell RNA-seq
SFRP1: Secreted frizzled related protein 1
SPP1: Secreted phosphoprotein 1
STAT5: Signal transducer and activator of transcription 5
SVM-RFE: Support vector machines-Recursive feature elimination
TGF-β1: Transforming growth factor-beta 1
TLR4: Toll-like receptor 4
TNF: Tumor necrosis factor
TREM2: Triggering receptor expressed on myeloid cells 2
UMIs: Unique molecular identifiers
VCAN: Versican.
