# Supplementary material for: NAMPT orchestrates fibroblast cuproptosis and immune crosstalk during IPF progression
Source: Front Immunol. 2026 May 20;17:1726692. doi: 10.3389/fimmu.2026.1726692 (PMC13229641; doi:10.3389/fimmu.2026.1726692)
Supplement: Supplementary file 1 [file DataSheet1.docx]

**
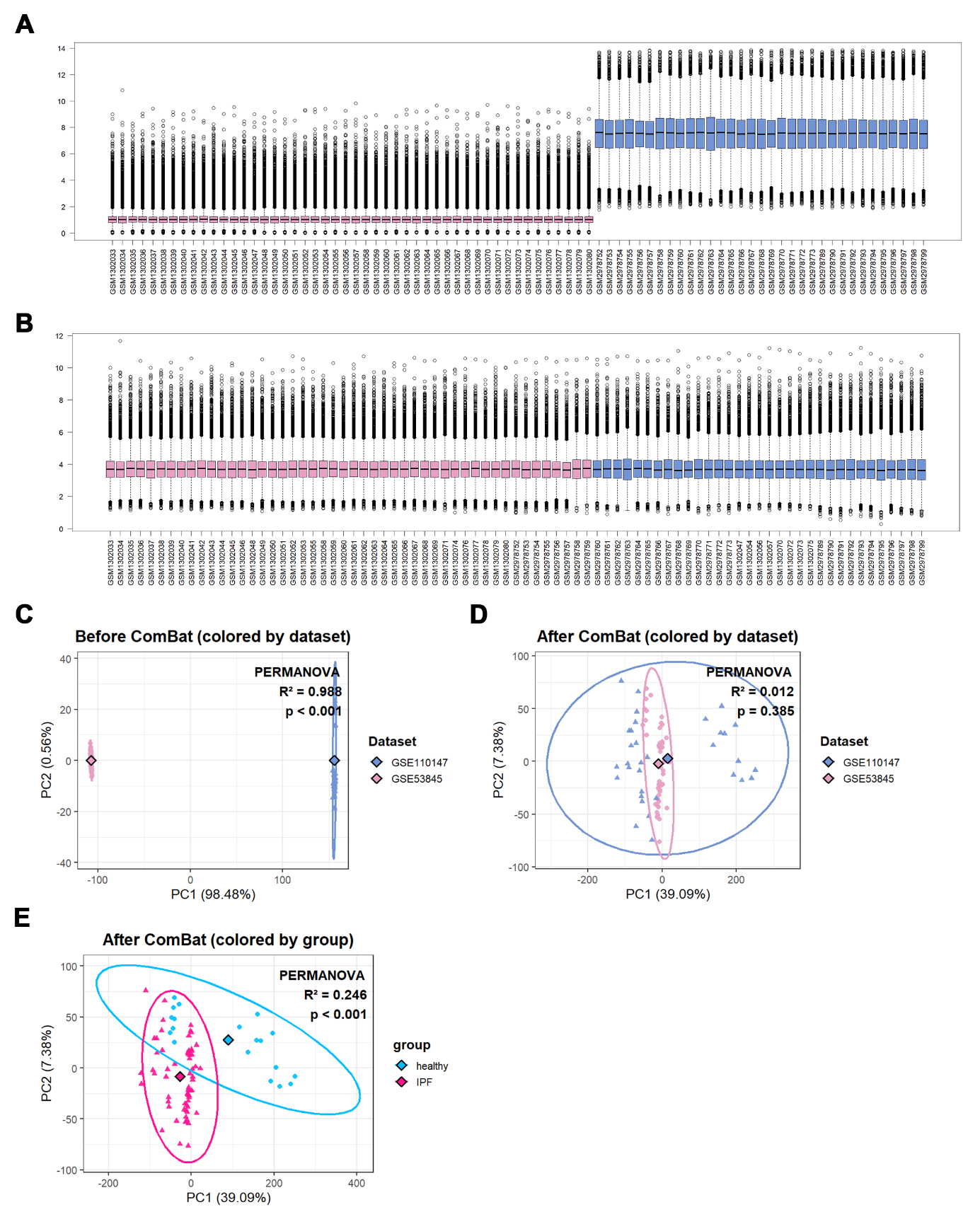
Figure S1. Assessment of batch-effect correction in the integrated bulk RNA-seq datasets.**

**Fig S1**

(A, B) Boxplots of sample-wise gene expression distributions before and after ComBat adjustment.

(C, D) PCA plots colored by dataset before and after batch correction, with PERMANOVA results indicating a marked reduction in dataset-associated variance after ComBat adjustment.

(E) PCA plot colored by disease status after batch correction, showing that disease-associated biological variation was preserved.


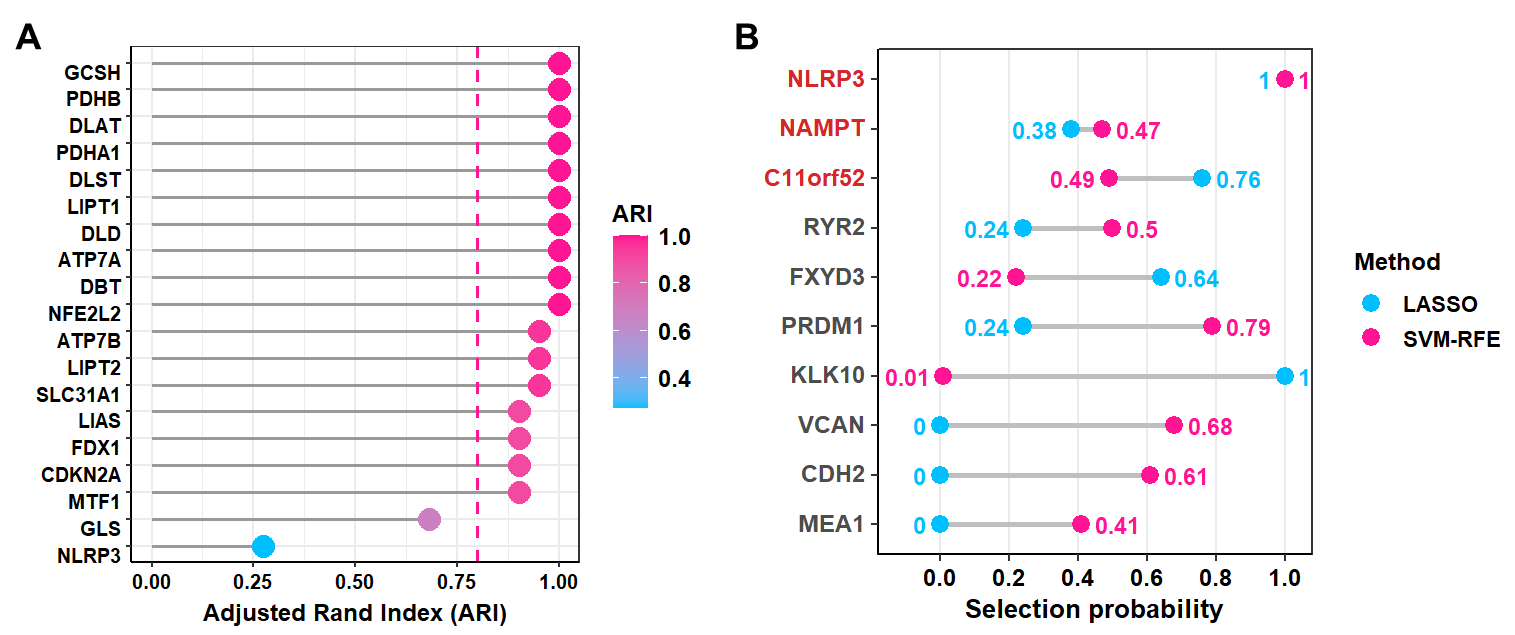


**Fig S2**

**Figure S2. Stability analyses for consensus clustering and hub gene selection.**

(A) Leave-one-gene-out analysis of consensus clustering based on the 19 CRGs. The adjusted Rand index (ARI) was calculated by comparing each perturbed clustering result with the original subtype assignment.

(B) Selection probabilities of the top candidate genes across repeated LASSO and SVM-RFE analyses. Genes with relatively high selection probabilities and low inter-method dispersion were considered more stable candidates.


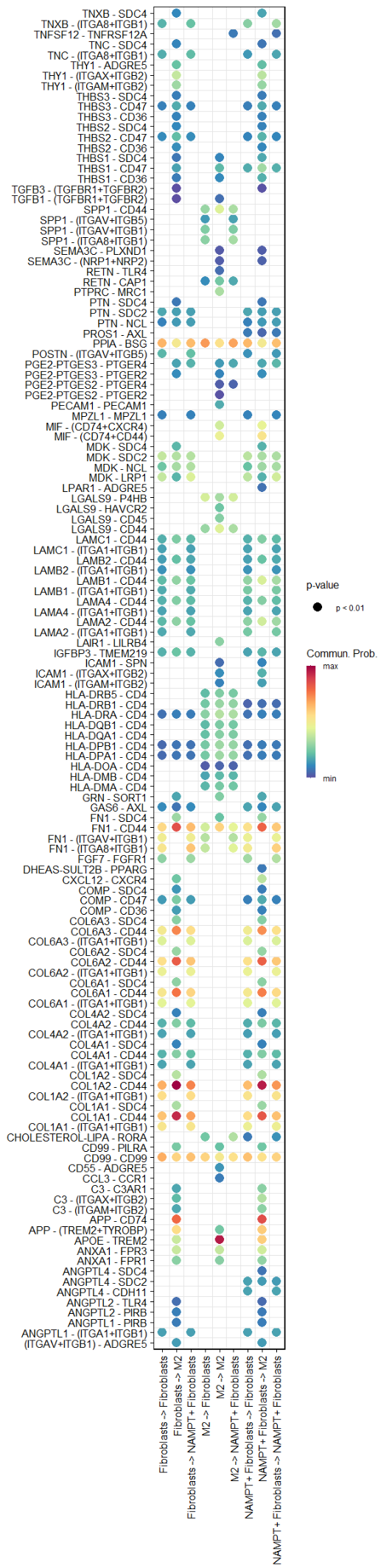


**Fig S3**

**Fig. S3** **Dot plot showing predicted ligand–receptor interactions between NAMPT+/- fibroblast and M2 macrophages.** Dot size represents significance (p-value), and color scale indicates communication strength.


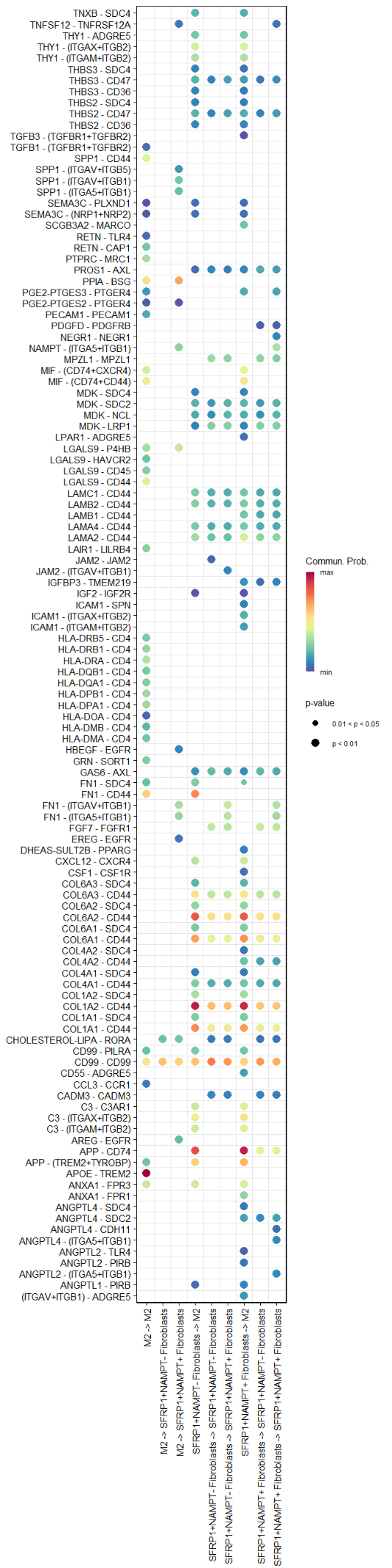


**Fig S4**

**Fig. S4** **Dot plot illustrating the predicted ligand–receptor interactions between NAMPT+ fibroblast subpopulations and M2 macrophages.** Dot size indicates statistical significance (p-value), and dot color represents communication probability inferred by CellChat analysis.
