# Supplementary material for: NAMPT orchestrates fibroblast cuproptosis and immune crosstalk during IPF progression
Source: Front Immunol. 2026 May 20;17:1726692. doi: 10.3389/fimmu.2026.1726692 (PMC13229641; doi:10.3389/fimmu.2026.1726692)

NAMPT Orchestrates Fibroblast Cuproptosis and Immune Crosstalk during IPF Progression

Junyu Jiang[1†], Xinghe Liu[1†], Guo Yang[1, 2], Jia Xie[1], Weijiao Mou[1, 3], Yunfei Xiang[1], Tao Zhang[1], Wenyu Du[1], Qingsong Chen[1], Fating Zhou[1], Guangbin Huang[1*] and Dingyuan Du[1*]

1 Department of Trauma Surgery, Chongqing Key Laboratory of Emergency Medicine, Chongqing Emergency Medical Center, Central Hospital, School of Medicine, Chongqing University, Chongqing 400014, China.

2 Department of cardiology, Chongqing Emergency Medical Center, Chongqing 400014, China.

3 Department of endocrinology, Chongqing Emergency Medical Center, Chongqing 400014, China.

*Corresponding author Dingyuan Du: Department of Trauma Surgery, Chongqing Emergency Medical Center, Chongqing 400014, China. E-mail address: dudingyuan@qq.com.

*Corresponding author Guangbin Huang: Department of Trauma Surgery, Chongqing Emergency Medical Center, Chongqing 400014, China. E-mail address: hgbin563@163.com.

† These authors contributed equally to this work.

Supplementary Figure 3: uncropped gel images. Images used to construct the indicated figure panels are listed.

Extended Data Fig. 9B NAMPT

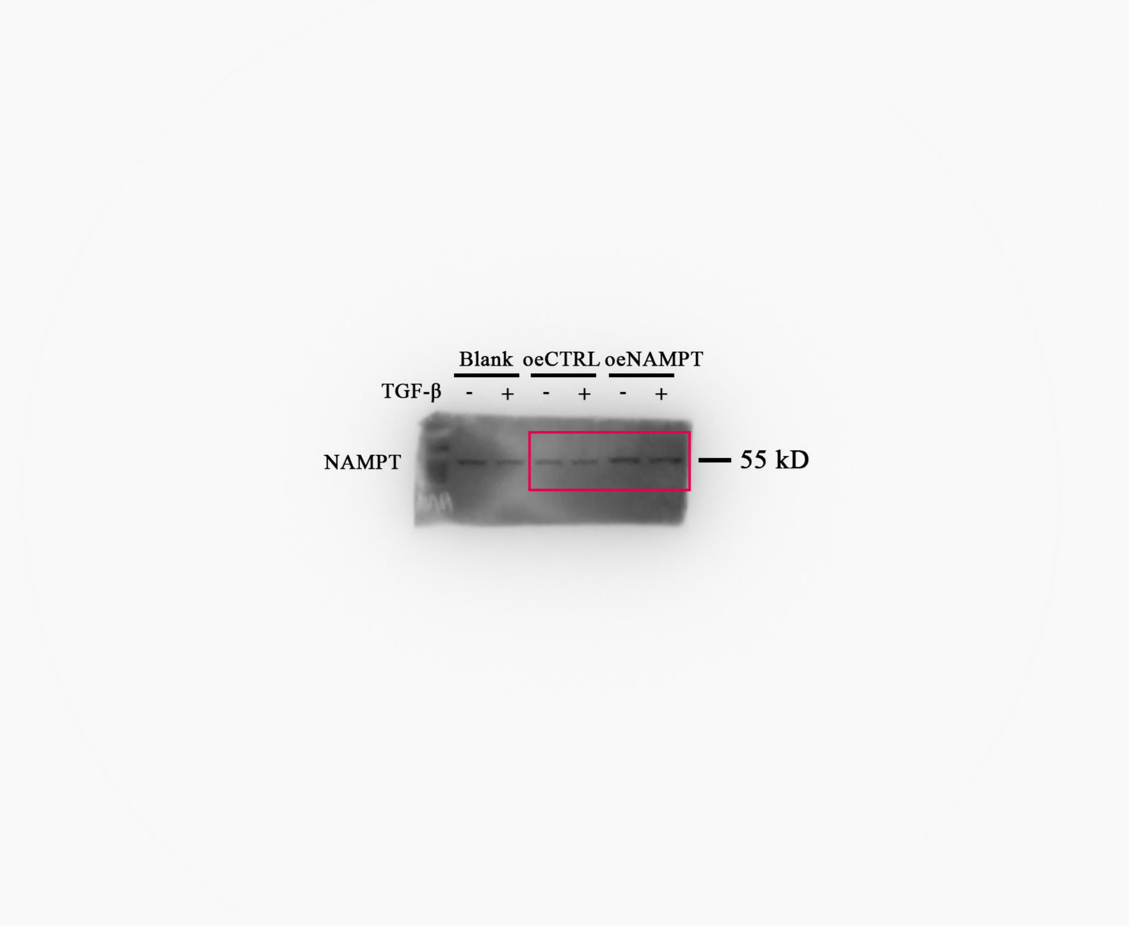

Extended Data Fig. 9B Flag

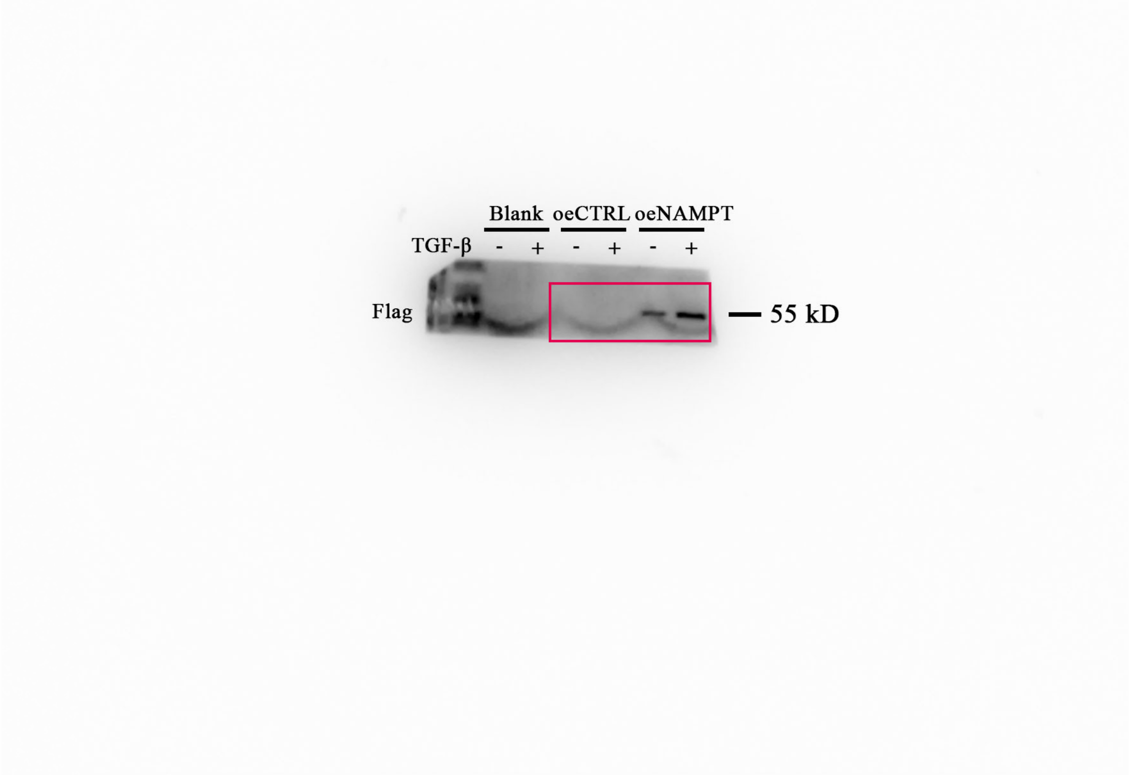

Extended Data Fig. 9B β-tubulin (NAMPT)

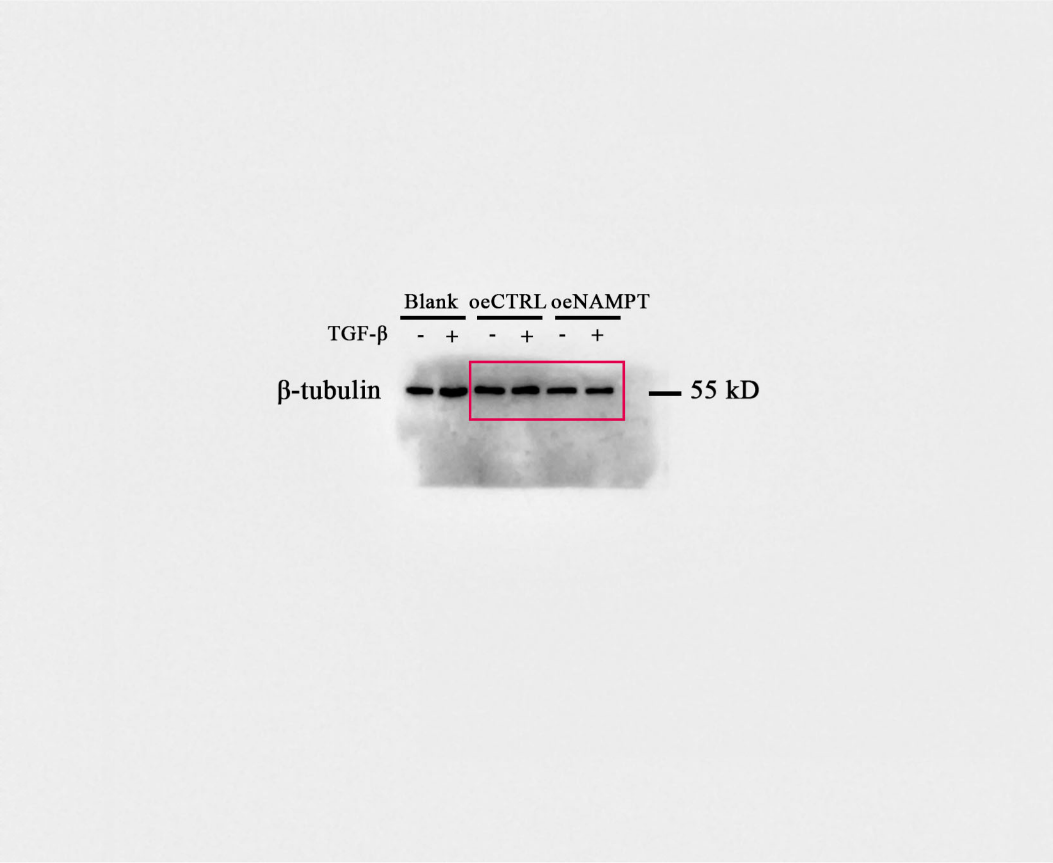

Extended Data Fig. 9B FDX1

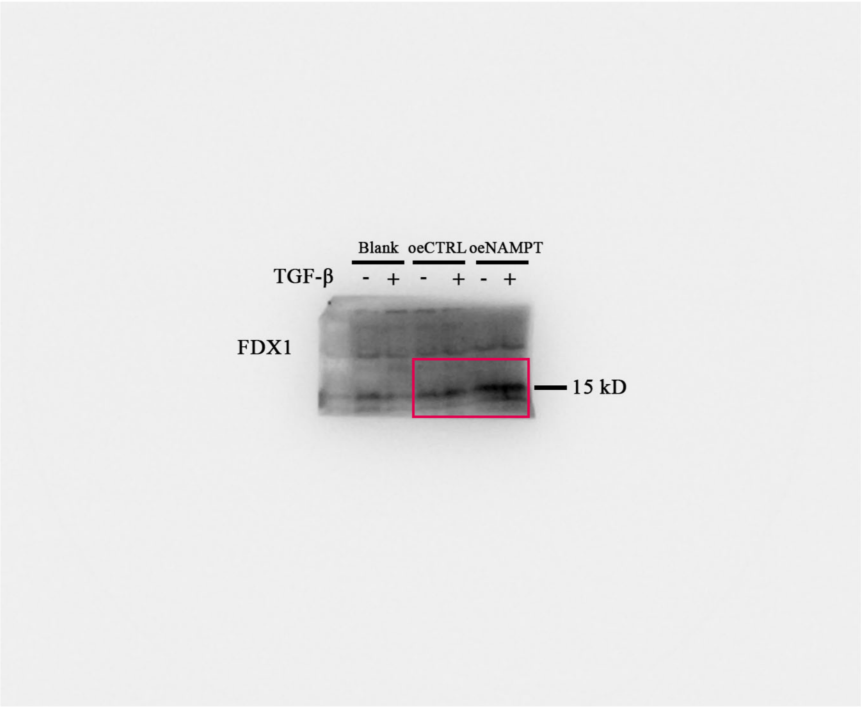

Extended Data Fig. 9B β-tubulin (FDX1)

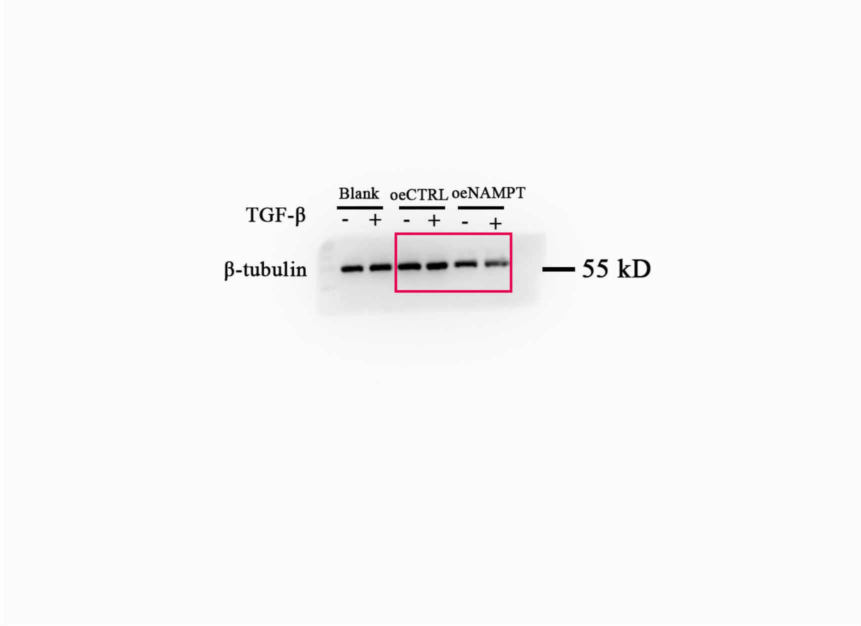

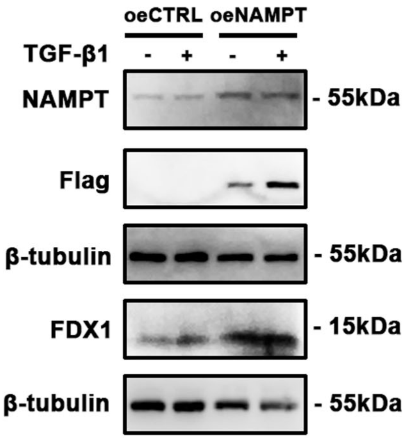

Extended Data Fig. 9C FDX1

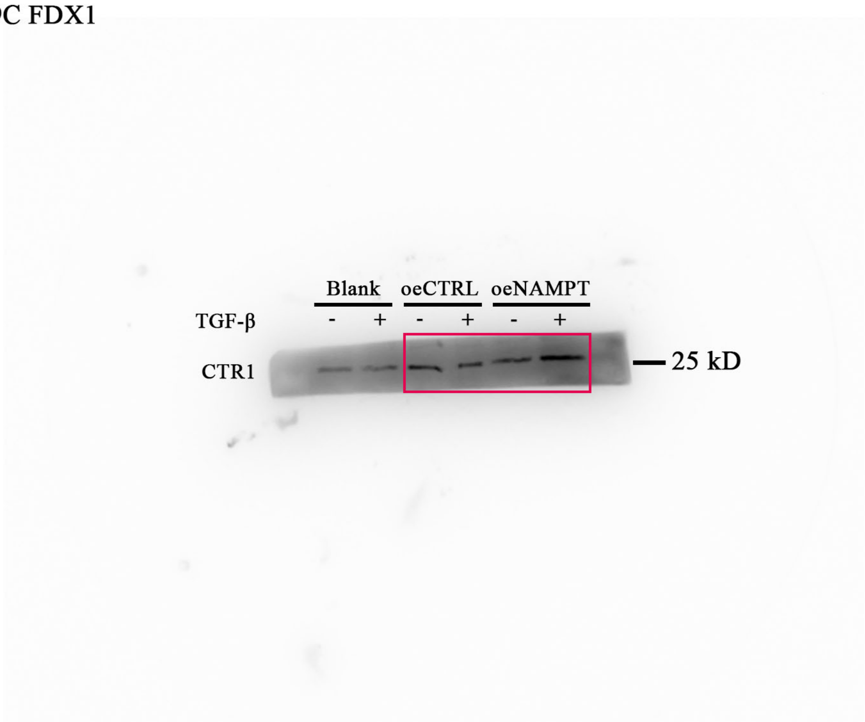

Extended Data Fig. 9C HSP70

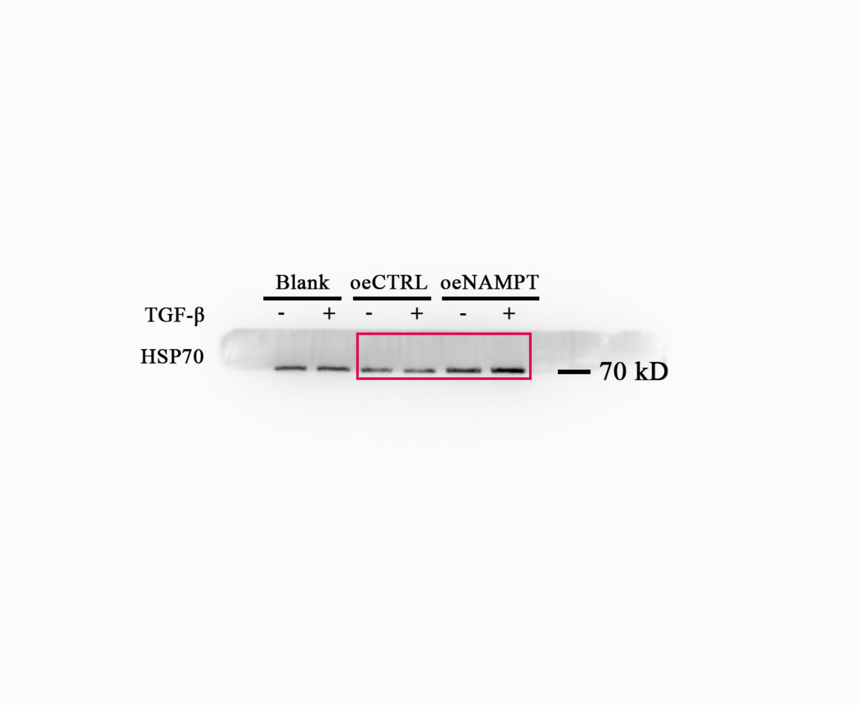

Extended Data Fig. 9C β-tubulin (CTR1 & HSP70)

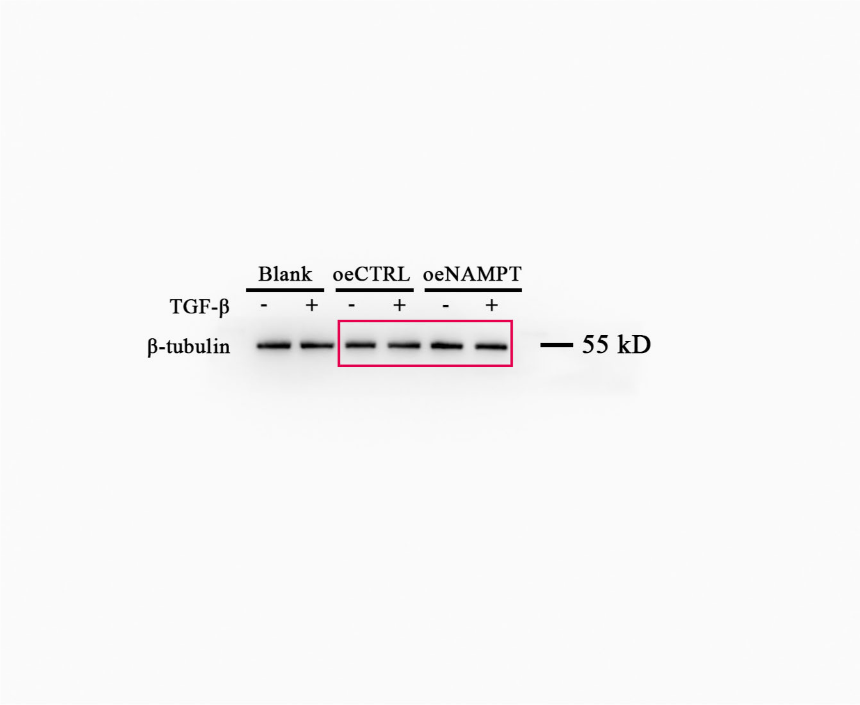

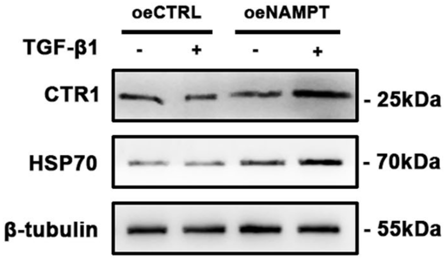

Supplement: Supplementary file 2 [file DataSheet2.pdf]
